# Developing a second-generation clinical candidate AAV vector for gene therapy of familial hypercholesterolemia

**DOI:** 10.1016/j.omtm.2021.04.017

**Published:** 2021-05-05

**Authors:** Lili Wang, Ilayaraja Muthuramu, Suryanarayan Somanathan, Hong Zhang, Peter Bell, Zhenning He, Hongwei Yu, Yanqing Zhu, Anna P. Tretiakova, James M. Wilson

**Affiliations:** 1 Gene Therapy Program, Department of Medicine, Perelman School of Medicine, University of Pennsylvania, Philadelphia, PA 19104, USA

**Keywords:** familial hypercholesterolemia, adeno-associated virus, gene therapy, LDLR, LDL, codon optimization, AAV8, HoFH, liver-directed

## Abstract

Gene therapy for hypercholesterolemia offers the potential to sustainably ameliorate disease for life with a single dose. In this study, we demonstrate the combinatorial effects of codon and vector optimization, which significantly improve the efficacy of an adeno-associated virus (AAV) vector in the low-density lipoprotein receptor (LDLR)-deficient mouse model (*Ldlr*^−/−^, *Apobec1*^−/−^ double knockout [DKO]). This study investigated vector efficacy following the combination of intervening sequence 2 (IVS2) of the human beta-globin gene and codon optimization with the previously developed gain-of-function, human LDLR triple-mutant variant (hLDLR-L318D/K809R/C818A) in the treatment of homozygous familial hypercholesterolemia (HoFH). Vector doses as low as 3 × 10^11^ genome copies (GC)/kg achieved a robust reduction of serum low-density lipoprotein cholesterol (LDL-C) by 98% in male LDLR-deficient mice. Less efficient LDL-C reduction was observed in female mice, which was attributable to lower gene transfer efficiency in liver. We also observed persistent and stable transgene expression for 120 days, with LDL-C levels being undetectable in male DKO mice treated with the second-generation vector. In conclusion, codon and vector optimization enhanced transgene expression and reduced serum LDL-C levels effectively at a lower dose in LDLR-deficient mice. The second-generation clinical candidate vector we have developed has the potential to achieve therapeutic effects in HoFH patients.

## Graphical abstract

**Figure:**
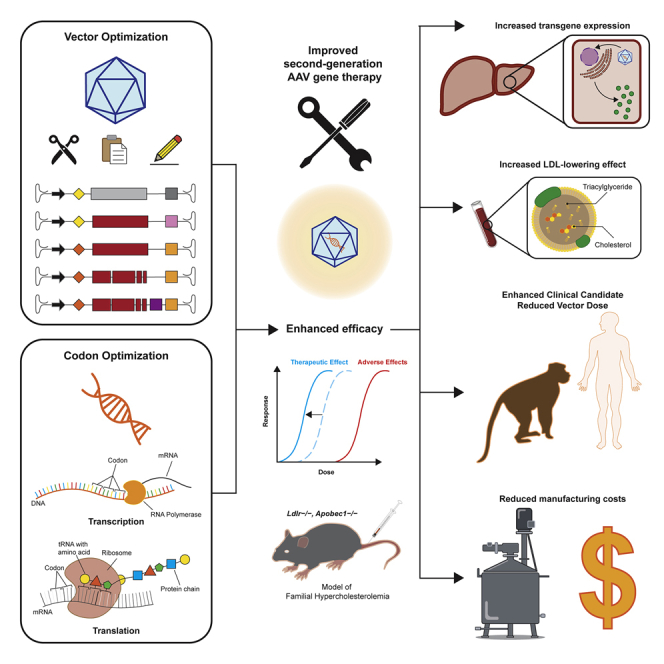

## Teaser

Combining codon and vector optimization significantly improved the efficacy of an AAV vector to treat a mouse model of homozygous familial hypercholesterolemia (HoFH). This second-generation clinical candidate vector has the potential to achieve therapeutic effects in HoFH patients and showcases translatable engineering strategies to enhance gene therapy vector efficacy.

## Introduction

Homozygous familial hypercholesterolemia (HoFH) is an autosomal-dominant monogenic disease that affects more than 30 million people worldwide.^1^ HoFH is primarily caused by mutations in three genes: low-density lipoprotein receptor (*LDLR*), apolipoprotein B100 (*APOB-100*), and proprotein convertase subtilisin/kexin type 9 (*PCSK9*).^2^ More than 1,200 molecular defects have been identified in the *LDLR* gene, with such mutations significantly impairing or ablating the receptor’s proper functionality. Homozygous patients with mutations affecting both alleles of the *LDLR* gene have very high low-density lipoprotein cholesterol (LDL-C) concentrations, exhibit premature coronary atherosclerosis, and develop cardiovascular disease before 30 years of age. Heterozygous familial hypercholesterolemia (HeFH) patients with one abnormal *LDLR* allele have clinical evidence of coronary artery disease (CAD) by 45 years of age.^3^

For most HoFH patients, the current lipid-lowering therapies, such as plasmapheresis, LDL apheresis, or liver transplantation, and cholesterol-lowering drugs (e.g., high-dose statins) are not able to reduce LDL-C to normal levels and also have adverse side effects.^4^, ^5^, ^6^ In recent years, PCSK9 inhibitors, such as evolocumab and alirocumab, have emerged as the newer generation of lipid-lowering drugs and can significantly reduce LDL-C in HeFH and non-familial hypercholesterolemia (non-FH) patients.^7^ However, the results have been variable in HoFH patients,^8^ and new therapeutic strategies are being sought. LDLR activity plays a vital role in mediating PCSK9 inhibitor efficacy, such that HoFH patients with a bi-allelic mutant *LDLR* gene or patients with residual LDLR activity <2% are considered receptor-negative subjects who lack any responses to PCSK9 inhibitor treatment.^8^ Gene therapy provides the possibility of stable and long-term expression of LDLR to correct the fundamental defect in HoFH.^9^ In recent years, liver-directed gene therapy by adeno-associated virus (AAV) vectors has shown encouraging efficacy in clinical trials for hemophilia A or B patients in which sustained therapeutic expression of clotting factor VIII or IX for up to 3 years was reported.^10^, ^11^, ^12^, ^13^ Based on efficacy data in preclinical studies in *LDLR*-deficient mouse models and pharmacological studies in nonhuman primates,^14^^,^^15^ the first-in-human clinical trial using AAV8.hLDLR for treatment in HoFH was started in 2016.^16^ However, one subject in cohort 1 [2.5 × 10^12^ genome copies (GC)/kg] and all three subjects in cohort 2 [7.5 × 10^12^ GC/kg] experienced an asymptomatic elevation in transaminases 4–6 weeks post-dosing and had positive T cell response to the AAV capsid.^17^ Efficacy data have not yet been reported.

The liver plays a crucial role in cholesterol homeostasis by regulating LDLR expression via a negative feedback mechanism that controls plasma cholesterol and maintains intracellular cholesterol levels.^18^ Hepatic *LDLR* is tightly regulated at the transcriptional level by sterol-regulatory element-binding protein (SREBPs), and at the post-transcriptional level by the PCSK9 and inducible degrader of LDLR (IDOL) pathways.^19^ SREBPs control the regulation of both *PCSK9* and *LDLR* expression, which primarily mediate the effects of statin treatment. Statins reduce LDL-C indirectly by increasing *LDLR* expression, but this effect is diminished by increased *PCSK9* expression.^20^^,^^21^ Based on human genetics studies and molecular mapping, the PCSK9-LDLR binding interface plays a crucial role in regulating circulating levels of LDL-C. PCSK9 has thus become a therapeutic target, with numerous clinical trials showing that PCSK9 inhibitors can safely reduce LDL-C levels up to 70%.^22^^,^^23^

We previously reported the development and evaluation of an AAV8 vector expressing a gain-of-function, human *LDLR* triple-mutant variant (hLDLR-L318D/K809R/C818A) in which this LDLR variant exhibited partial resistance to degradation via PCSK9 and IDOL pathways in mouse models of *LDLR* deficiency.^24^ The partial resistance to degradation in this gain-of-function LDLR variant was found to be dose dependent in the presence of human PCSK9 or IDOL overexpression.^24^ In this study, we aimed to develop a more efficient second-generation AAV vector for gene therapy of HoFH through codon and vector optimization. We report that the combination of codon and vector construct optimization increased the efficacy of the AAV.hLDLR vector by 10-fold. The combination of codon and vector optimization, the triple-mutant LDLR variant, and an efficient liver-tropic AAV capsid suggests that this vector has great potential to safely achieve clinical benefits in HoFH patients at a low dose.

## Results

### Improve LDLR expression by codon optimization

**Figure 1:**
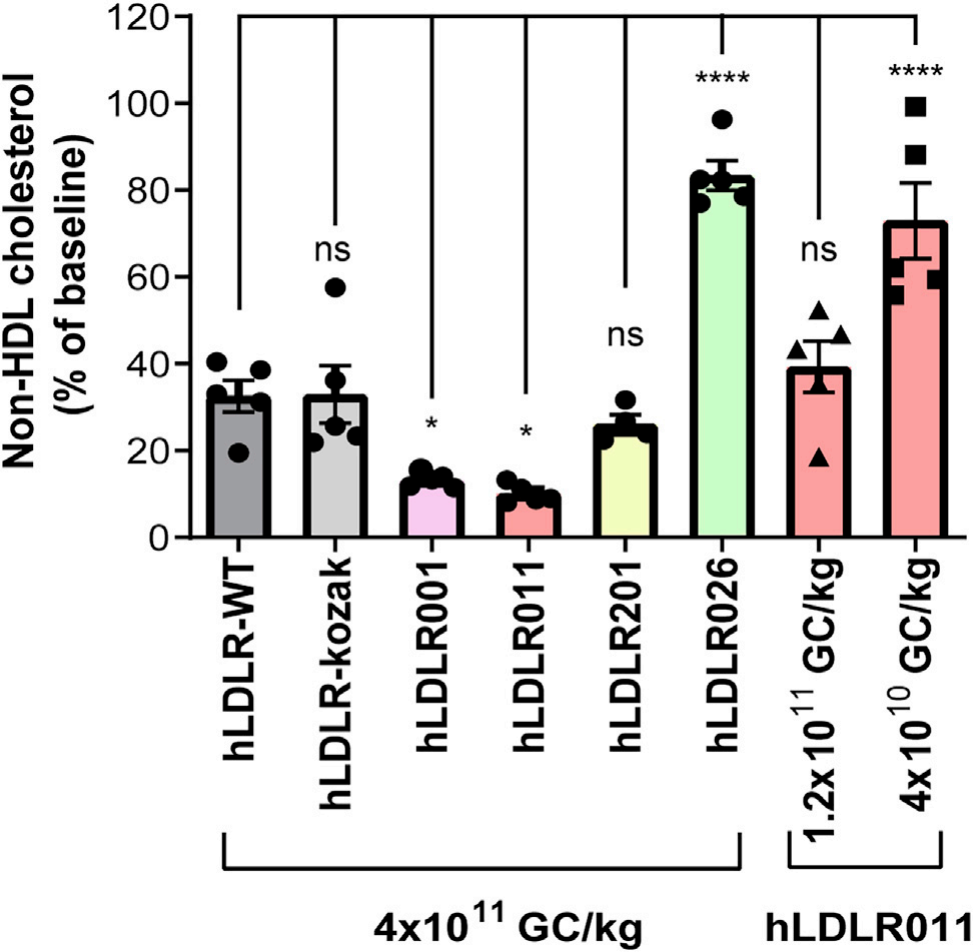
Evaluation of different codon-optimized hLDLR cDNA sequences in male DKO mice Serum non-HDL cholesterol levels in male DKO mice were reduced 14 days after intravenous administration of AAV8.hLDLR vectors. Serum non-HDL levels are shown as percentage of baseline levels. All six vectors were tested at 4 × 10^11^ GC/kg. LDLR011 was also evaluated at 1.2 × 10^11^ and 4 × 10^10^ GC/kg. Individual mouse data (five mice per group) and means ± SEM are shown. Each group was compared to the hLDLR-WT group. ∗p < 0.05, ∗∗∗∗p < 0.0001, one-way ANOVA followed by Dunnett’s multiple comparison test.

To improve expression levels of human *LDLR* in the AAV vector, we first incorporated the Kozak sequence in front of the start codon; the native h*LDLR* sequence (wild type [WT]) does not contain the Kozak consensus sequence, which has been shown to enhance translation from the correct initiation codon.^25^ When comparing the two vectors in *Ldlr*^−/−^, *Apobec1*^−/−^ double-knockout (DKO) mice, however, the two groups of mice showed similar levels of non-HDL-C reduction 14 days after vector treatment (Figure 1). Because codon optimization has been shown to increase transgene expression levels in AAV vectors,^26^ we designed four codon-optimized h*LDLR* cDNA sequences that also contain the Kozak consensus sequence to replace the WT h*LDLR* cDNA sequence in the AAV vector and packaged each construct in AAV8 capsids for *in vivo* evaluation in male DKO mice. Some properties of the cDNA, such as codon adaptation index (CAI), guanine-cytosine (GC)-content, rare codon percentage, and codon frequency distribution, are shown in Figure S1.

To determine which codon-optimized hLDLR reduced serum non-HDL-C more efficiently, we performed a pilot study that involved injecting 4 × 10^11^ GC/kg AAV8-hLDLR vectors intravenously in male DKO mice. Fourteen days after vector treatment, serum non-HDL-C levels reduced: AAV8.hLDLR-WT by 68%, hLDLR-Kozak by 67%, hLDLR001 by 87%, hLDLR011 by 90%, hLDLR201 by 74%, and hLDLR026 by 17% (Figure 1). The hLDLR026 variant showed significantly less efficient reduction compared with hLDLR-WT, thereby indicating that not all codon optimization necessarily leads to improved expression. We further evaluated the dose effect of hLDLR011 at two lower doses, 1.2 × 10^11^ and 4 × 10^10^ GC/kg. hLDLR011 did not show efficient reduction at 4 × 10^10^ GC/kg. At 1.2 × 10^11^ GC/kg, hLDLR011 showed 61% reduction of LDL-C, similar to hLDLR-WT at 4 × 10^11^ GC/kg (Figure 1), suggesting hLDLR011 is about 3-fold more efficient than hLDLR-WT. Based on the pilot study data, we proceeded with hLDLR011 as the cDNA sequence for further vector optimization. In addition, we introduced the previously demonstrated gain-of-function triple mutations (hLDLR-L318D/K809R/C818A) to confer partial resistance to LDLR degradation via the PCSK9 or IDOL pathway to the hLDLR011 cDNA sequence to generate hLDLR011-T.^24^

### Improve LDLR expression by vector optimization

**Figure 2:**
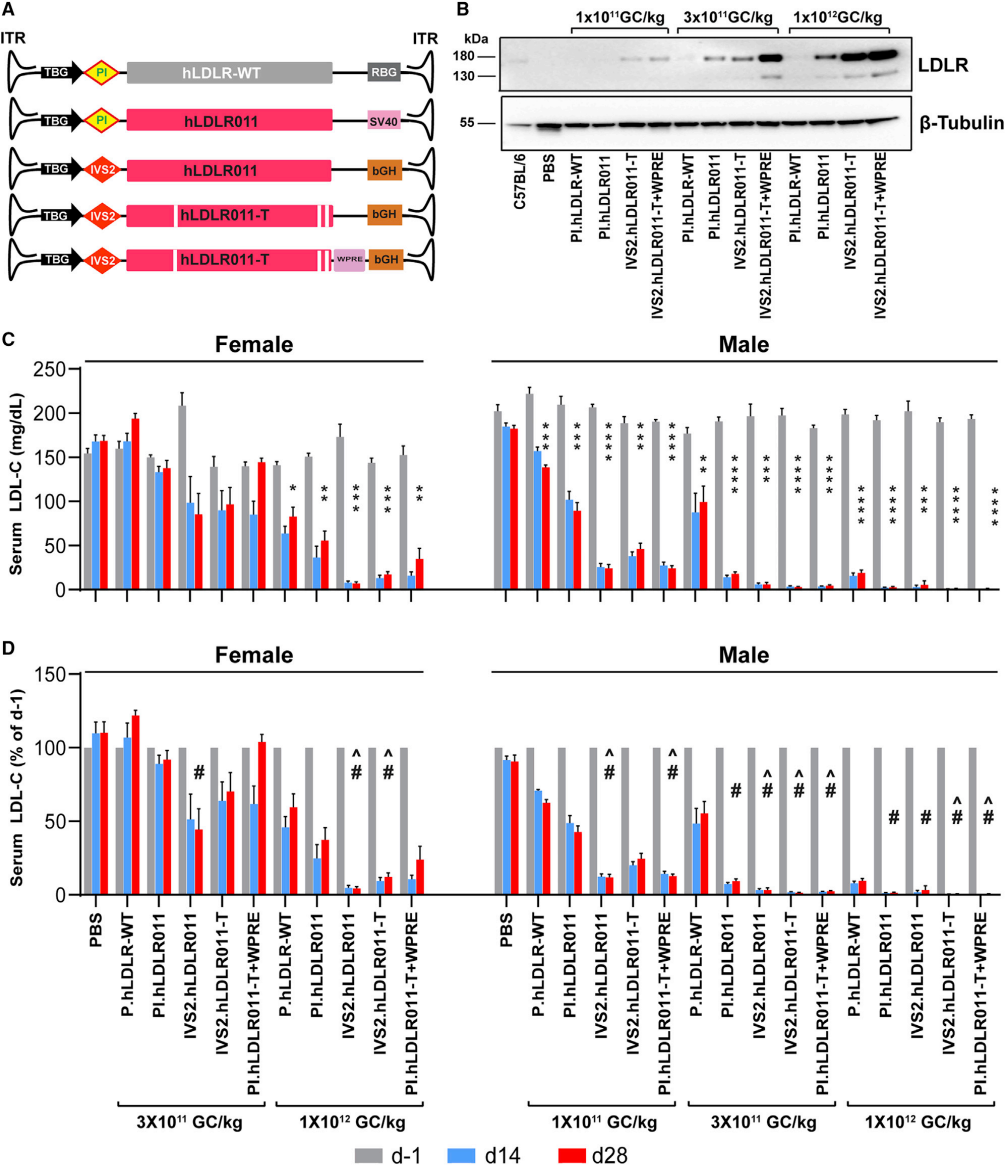
Vector optimizations improve efficacy in DKO mice (A) Schematic illustration of different AAV.hLDLR vector constructs. (B) Western blot analysis on liver lysates (20 μg/lane) from male DKO mice 28 days post-administration of PBS or AAV8.hLDLR vectors at 1 × 10^11^, 3 × 10^11^, and 1 × 10^12^ GC/kg. Liver lysate from an untreated C57BL/6 mouse was included as a reference. One representative sample from each group is shown. (C) Serum LDL-C levels at 1 day before (d−1) or 14 and 28 days (d14 and d28, respectively) after administration of PBS or AAV8.hLDLR vectors at the indicated doses in female and male DKO mice expressed as mg/dL. The significance of reduction on d28 compared with d−1 is indicated. Mean ± SEM are shown (n = 5/group). ∗p < 0.05, ∗∗p < 0.01, ∗∗∗p < 0.001, ∗∗∗∗p < 0.0001. One-way ANOVA followed by Dunnett’s multiple comparison test. Detailed statistical differences after treatment on d14 and d28 are shown in Table S1. (D) Serum LDL-C levels shown as percentage of levels at d−1. Means ± SEM are shown (n = 5/group). Vectors with d28 serum LDL-C (% of d−1) levels significantly lower than the levels in PI.hLDLR-WT (#) or PI.hLDLR011 (ˆ), respectively, within the same dose and sex cohorts. One-way ANOVA followed by Tukey’s multiple comparison test. Detailed statistical differences between different vectors on d14 and d28 are shown in Table S2. bGH, bovine growth hormone polyA; hLDLR011-T, LDLR011 carrying the triple-mutant variant (hLDLR-L318D/K809R/C818A); IVS2, intervening sequence 2 of human beta-globin gene; PI, Promega intron; RBG, rabbit beta-globulin polyA; SV40, SV40 polyA; WPRE, woodchuck hepatitis virus post-transcriptional regulatory element.

**Figure 3:**
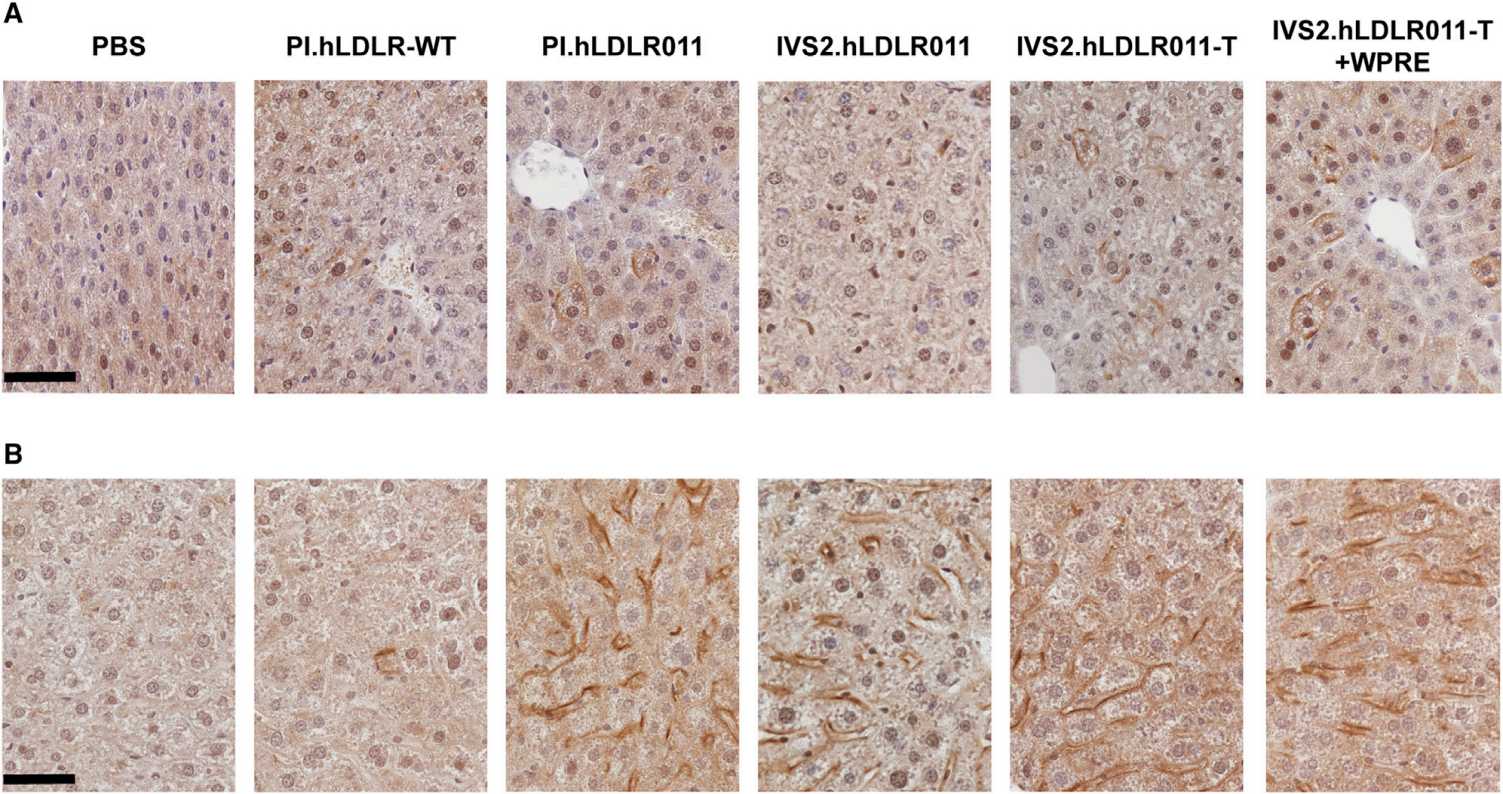
Vector optimizations improve LDLR protein expression in DKO mice (A and B) Livers were harvested 28 days after AAV8.hLDLR vector administration at 3 × 10^11^ GC/kg in female (A) and male (B) DKO mice for immunohistochemical staining for hLDLR. Representative images are shown. Scale bar, 50 μm.

To further improve h*LDLR* expression from AAV vectors containing the liver-specific thyroxine-binding globulin (TBG) promoter, we evaluated the effects of a different intron sequence, intervening sequence 2 (IVS2) of the human beta-globin gene or the WPRE (woodchuck hepatitis virus post-transcriptional regulatory element) at the 3′ end of the cDNA in the expression cassette (Figure 2A). These two elements have been previously used in AAV vectors for hemophilia B gene therapy and achieved efficient clotting factor IX expression.^27^^,^^28^ We then compared the efficacy of the hLDLR-WT and four of the optimized hLDLR011 vectors in both female and male DKO mice at multiple vector doses. Liver tissues were harvested from male DKO mice on day (d) 28 post-vector administration for Western blot analyses to evaluate hLDLR protein levels in liver lysate. Vector- and dose-dependent effects on LDLR protein expression were observed. PI.hLDLR-WT (vector currently being used in the clinical trial ClinicalTrials.gov: NCT02651675) hardly showed any detectable signals even at the highest dose tested (1 × 10^12^ GC/kg), while hLDLR011 vectors containing IVS2 or WPRE showed LDLR signals at the lowest dose tested (1 × 10^11^ GC/kg) (Figure 2B). We measured serum LDL-C levels in mice 1 day before dosing (d−1), as well as 14 and 28 days after vector dosing, and levels are shown in Figure 2C. In female DKO mice, none of the 3 × 10^11^ GC/kg dose groups showed significant reduction of serum LDL-C at d14 or d28 after dosing (Figure 2C; Table S1). However, when comparing normalized LDL-C levels (percentage of d−1) between different vectors at the 3 × 10^11^ GC/kg dose in females, the two IVS2-containing vectors performed significantly better than the other groups (Figure 2D; Table S2). At 1 × 10^12^ GC/kg, all vectors significantly reduced serum LDL-C levels in females (Figure 2C; Table S1), with the two IVS2-containing vectors showing more significant reduction than the other three vectors (Figure 2D; Table S2). Male DKO mice showed more robust reduction compared with female mice, consistent with our previous observations in DKO mice using the murine *LDLR* or human *LDLR* cDNA.^14^ Male mice treated with hLDLR011 vectors at 3 × 10^11^ GC/kg showed 93%–98% reduction of LDL-C on d14 after vector treatment, while the hLDLR-WT vector-treated mice showed 52% of reduction (Figure 2D). At 1 × 10^12^ GC/kg dose, LDL-C levels in hLDLR011 vector-treated male mice were almost undetectable. We therefore treated the males with a lower dose (1 × 10^11^ GC/kg) and found that vectors containing IVS2 or WPRE showed enhanced LDL-C reduction compared with PI.h*LDLR*011 alone (Figure 2D; Table S2). Immunohistochemical (IHC) staining of hLDLR in liver harvested at d28 after vector treatment showed strong staining on the lining of most hepatocytes in male DKO mice treated with hLDLR011 or hLDLR011-T vectors (Figure 3B) compared with the fewer positive hepatocytes in female DKO mice (Figure 3A).

### More efficient gene transfer in the liver of male DKO mice than in female DKO mice

**Figure 4:**
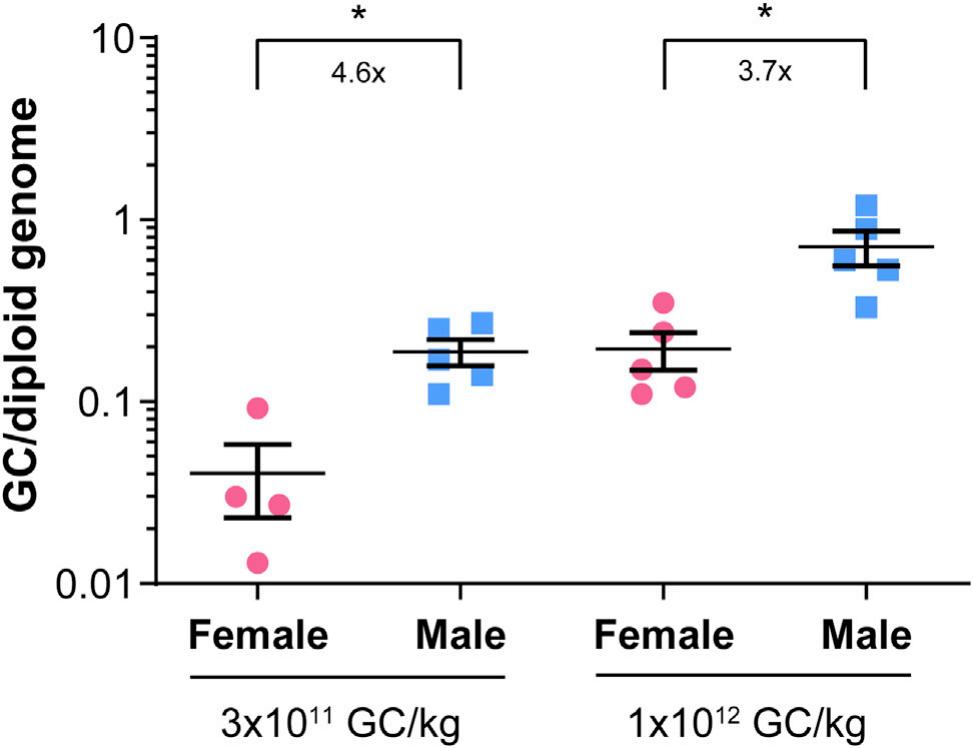
Gene transfer is more efficient in male DKO mice than in female DKO mice Liver was harvested 28 days after AAV8.IVS2.hLDLR011-T vector administration at 3 × 10^11^ and 1 × 10^12^ GC/kg in female and male DKO mice for DNA isolation followed by qPCR. Individual mouse data and means ± SEM are shown. Statistical differences between female and male at each dose were analyzed by unpaired two-tailed Student’s t test.

In one of our previous studies to evaluate AAV8.TBG.PI.hLDLR-WT vector biodistribution in DKO mice at the dose of 7.5 × 10^12^ GC/kg, male mice showed slightly higher vector genome copies in liver than the female mice. However, these differences were only significant at a later time point of d180, but not on d3, d14, and d90.^29^ To determine whether gene transfer efficiency was affected by sex in the DKO mice in this study when vector doses were 7.5- to 25-fold lower, we measured vector genome copies in d28 liver samples from male and female mice treated with IVS2.h*LDLR*011-T at two vector doses (3 × 10^11^ and 1 × 10^12^ GC/kg). Male mice had significantly higher (3- to 5-fold higher) vector genome copies in the liver than the female mice at both doses (Figure 4). Males treated with 3 × 10^11^ GC/kg had similar vector genome copies as the female mice treated with 1 × 10^12^ GC/kg, suggesting AAV8 gene transfer was about 3-fold higher in male DKO mice than in female mice. Mice with vector genome copies in liver as low as 0.2 copies per diploid genome (female at 1 × 10^12^ GC/kg and male at 3 × 10^11^ GC/kg, respectively) demonstrated efficient reduction of LDL-C at 90%–98% (Figures 2B and 2C).

### Enhancing h*LDLR* mRNA levels by IVS2 and WPRE

**Figure 5:**
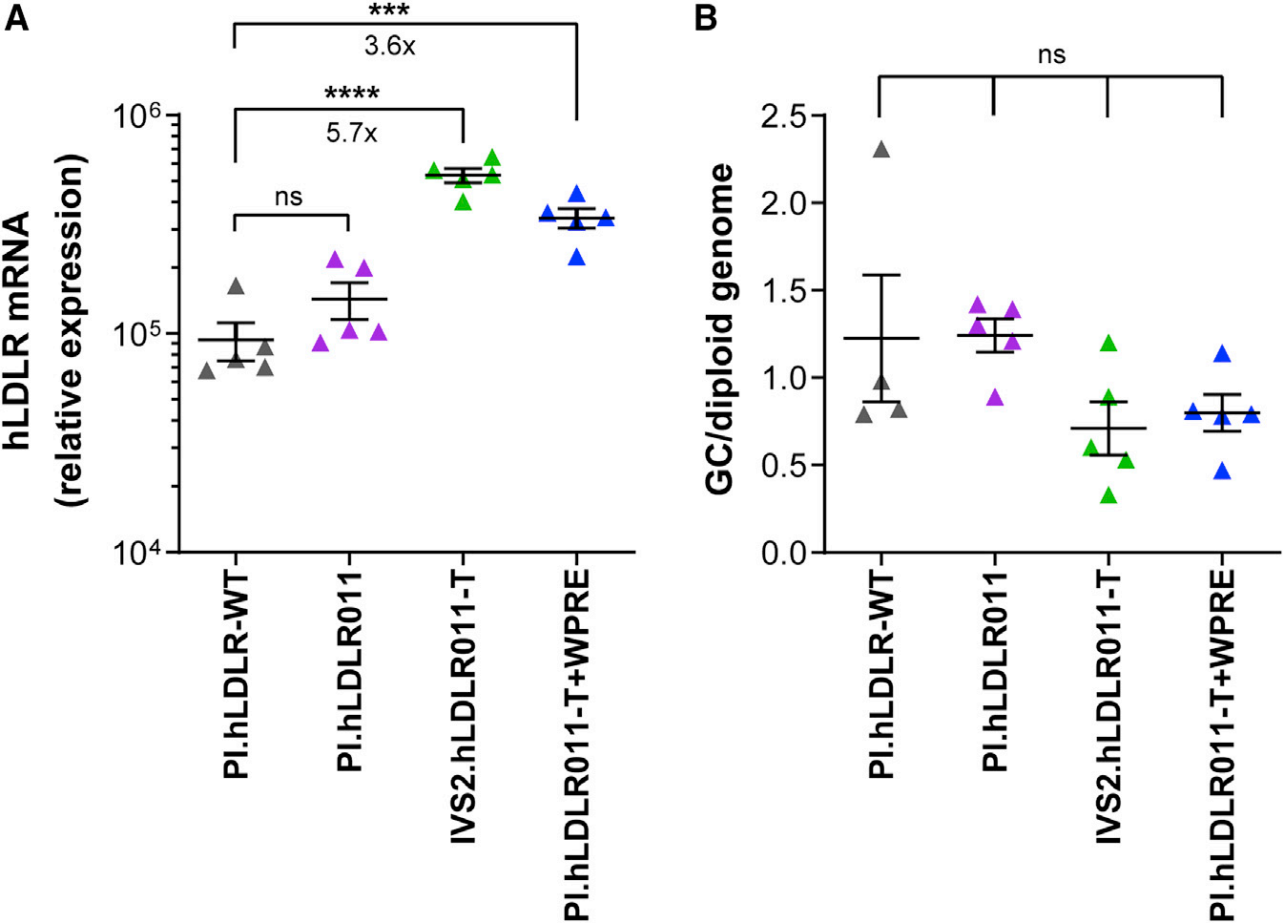
Vector optimization improves LDLR mRNA levels in male DKO mice (A and B) Livers were harvested 28 days after AAV8.hLDLR vector administration at 1 × 10^12^ GC/kg in male DKO mice for RNA isolation followed by qRT-PCR (A) or DNA isolation for vector GC analysis by qPCR (B), respectively. Individual mouse data and means ± SEM are shown. ∗∗∗p < 0.001, ∗∗∗∗p < 0.0001, one-way ANOVA followed by Tukey’s multiple comparison test.

To confirm the roles of IVS2 and WPRE in enhancing h*LDLR* mRNA levels, we compared the h*LDLR* mRNA levels in the liver of male DKO mice treated with 1 × 10^12^ GC/kg hLDLR vectors. IVS2-containing vectors showed 5.7-, 3.6-, and 1.6-fold higher *LDLR* mRNA levels than Promega intron (PI)-hLDLR-WT, PI.hLDLR011, and PI.hLDLR011-T + WPRE, respectively (Figure 5A). The WPRE-containing vector showed 3.6- and 2.4-fold higher *LDLR* mRNA levels than h*LDLR*-WT and PI.hLDLR011, respectively (Figure 5A); these data are consistent with previous reports on the function of WPRE.^30^, ^31^, ^32^ Vector genome copy analysis by quantitative PCR (qPCR) showed no statistical differences between the mice treated with different vectors (Figure 5B), indicating equivalent gene transfer efficiency by the four vectors, and the increased mRNA levels were attributed by IVS2 or WPRE. The size limitation of the AAV vector does not allow the h*LDLR* vector to contain both IVS2 and WPRE; we have previously reported the development and evaluation of gain-of function, human *LDLR* triple-mutant variant (hLDLR-L318D/K809R/C818A), which exhibits partial resistance to degradation via PCSK9 and IDOL pathways.^24^ Therefore, we selected IVS2.hLDLR011-T for further evaluation.

### Kinetics of LDLR expression in male DKO mice

**Figure 6:**
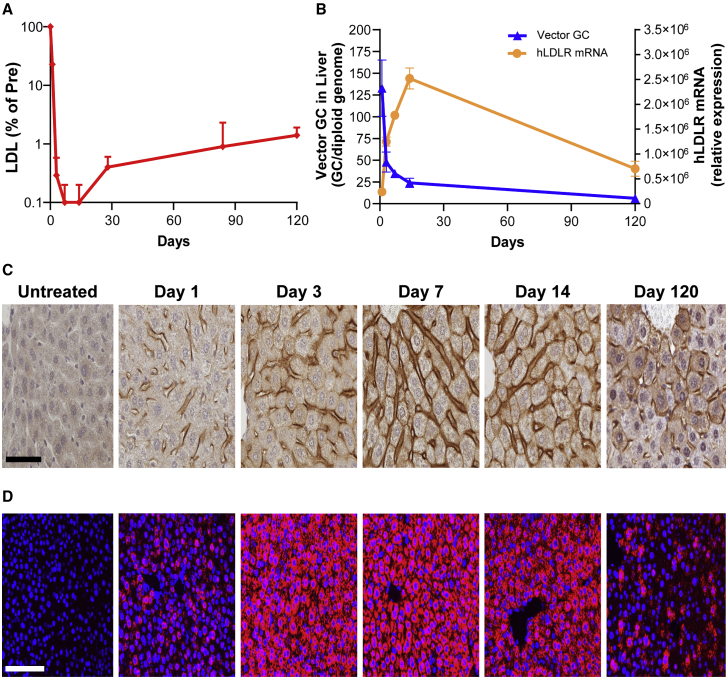
Kinetics of hLDLR expression in male DKO mice Male DKO mice received 7.5 × 10^12^ GC/kg AAV8.IVS2.hLDLR-T via tail vein injection. Serum LDL-C levels were monitored over the course of the study. Livers were harvested from a cohort of five mice on d1, d3, d7, d14, and d120 for analysis. (A) Serum LDL-C levels. Means + SEM are shown (n = 5/time point). (B) Vector genome copies in liver measured by qPCR and hLDLR mRNA levels in liver measured by qRT-PCR. Means ± SEM are shown (n = 5/time point).(C) IHC to detect hLDLR protein. Scale bar, 50 μm. (D) ISH to detect vector DNA and hLDLR mRNA. Representative images are shown. Scale bar, 100 μm.

To evaluate the kinetics of the IVS2.hLDLR011-T vector, we dosed male DKO mice with AAV8.IVS2.hLDLR011-T vector at 7.5 × 10^12^ GC/kg, the same dose being administered to cohort 2 in the current AAV8 gene therapy trial for HoFH.^16^ One day after dosing, LDL-C levels were quickly reduced by 77% and reached a barely detectable level by d3 that persisted for the remainder of the study duration (d120) (Figure 6A). The robustness of LDL-C reduction was not achieved by PI.hLDLR-WT, the first-generation FH gene therapy vector.^14^^,^^33^ We harvested liver tissue (n = 5 mice/time point) on d1, d3, d7, d14, and d120 after vector administration. Vector genome copies in the liver measured by qPCR were high at d1 post-dosing, but then declined over time (Figure 6B). We detected a 4-fold reduction of vector genome in liver between d14 and d120. On the other hand, h*LDLR* mRNA expression increased from d1 to d14 before decreasing by 3.6-fold on d120, a rate similar to the vector DNA reduction (Figure 6B). Liver samples collected at each time point were also examined by IHC staining and *in situ* hybridization (ISH) for detection of LDLR protein and h*LDLR* DNA and mRNA in liver. Both LDLR protein and mRNA were detectable 1 day after dosing and kept increasing until d7–d14 (Figures 6C and 6D). Both IHC and ISH signals were significantly reduced on d120, consistent with the qPCR results (Figure 6B).

## Discussion

The successful clinical trial of liver-directed gene transfer for hemophilia B^34^ paved the way for several clinical studies with AAV liver-directed gene transfer in recent years. It has been demonstrated that not only plasma protein deficiencies but also metabolic disorders can be treated with liver-directed gene therapy.^35^ However, the potential clinical ramifications of AAV gene therapy should be approached very cautiously. Dose-correlated efficacy, host immune response, and toxicity have been reported in several clinical trials in which AAV vector was delivered systemically.^36^^,^^37^ All three patients in cohort 2 [7.5 × 10^12^ GC/kg] of the first AAV8-mediated h*LDLR* gene transfer trial in HoFH patients (ClinicalTrials.gov: NCT02651675) experienced an asymptomatic elevation in transaminases 4–6 weeks post-dosing.^17^ For genetic diseases such as HoFH, an ideal gene therapy vector should be able to achieve sustained therapeutic effects at a low or tolerable vector dose without eliciting harmful immune responses or toxicity. The combination of an AAV serotype with high transduction efficiency and an optimized vector construct could potentially achieve therapeutic effects at a safe vector dose. In this study, we aimed to develop a second-generation human LDLR transgene expression cassette with improved expression levels that could ultimately be used to treat HoFH patients.

Codon optimization has become a commonly used method to increase the expression of biotherapeutic recombinant proteins, such as antibodies. Increased expression of an antibody can be obtained by using synonymous codon mutations to fine-tune the expression of one of two light chain genes of a bispecific antibody.^38^ Recently, it was shown that synonymous codon usage in open reading frame (ORF) sequences determines both gene expression levels and translation dynamics, suggesting codon usage influences gene expression.^39^, ^40^, ^41^

In 2011, Nathwani et al.^34^ achieved sustained therapeutic expression of clotting factor IX in six human patients with severe hemophilia B by injecting a single dose of self-complementary AAV8 vector expressing a codon-optimized human factor IX transgene cDNA. Following this successful clinical trial, several pharmaceutical companies have used alternative AAV vector serotypes and different codon-optimized transgenes in many clinical trials. Similarly, we have developed variants of codon-optimized h*LDLR* cDNA sequences to achieve maximum expression at a low vector dosage. Our study highlighted that hLDLR011 is 3-fold more efficient than WT human *LDLR*. In a variety of organisms, transcriptional efficiency has been increased because of the occurrence of IVSs (or introns) at their 5′ UTR. One of the key early studies demonstrated that recombinant simian virus 40 carrying β-globin cDNA showed 400-fold increased RNA production in the presence of either β-globin IVS1 or IVS2 in their expression cassette compared with the intron-deficient transgene vector.^42^ The inclusion of IVS2 or WPRE in the expression cassette increased expression of clotting factor IX, with this approach being employed effectively in AAV gene therapy vectors used to treat hemophilia B.^27^^,^^28^ We have also demonstrated that the incorporation of IVS2 or WPRE increased the transgene expression several-fold compared with PI.hLDLR-WT or PI.hLDLR011.

In this study, we observed that at a low dose of 3 × 10^11^ GC/kg dose, male DKO mice treated with AAV8.IVS2.hLDLR011-T vector had about 0.2 vector genome copy per diploid genome but showed strong expression of hLDLR on the surface of transduced hepatocytes (Figure 3B), which resulted in 98% reduction of LDL-C 14 days after vector treatment (Figure 2C). This indicates that broad transduction and expression of LDLR in the liver may not be essential for achieving therapeutic effects in HoFH. Strong expression of LDLR in a few transduced hepatocytes could achieve dramatic therapeutic effects in a mouse model of HoFH.

We also observed the influence of sex on liver-directed AAV transduction efficiency in mice. In our study, female DKO mice had 4- to 5-fold lower vector genome copies in liver at the same doses. This impact of sex on liver transduction efficiency in mouse has been previously reported by other groups.^43^, ^44^, ^45^ AAV transgene expression is reduced in male mice to levels seen in female mice following the castration of male mice, thereby suggesting that androgens play a vital role in transgene expression within hepatocytes.^43^ However, the influence of sex on liver-directed gene transfer in nonhuman primates is not evident.^15^

*LDLr*^−/−^ *Apobec*^−/−^ DKO mice display a substantial increase of plasma LDL-C levels on standard chow and develop severe atherosclerosis, making this a more suited model to study the pathophysiology of human FH.^46^ The DKO mice develop severe spontaneous atherosclerosis slowly and progressively on a standard chow diet as they age.^47^ At the age of 12 weeks, a fatty streak develops in the proximal aortic region, and with age, plaque formation worsens. Severe plaque formation occurs by the age of 72 weeks, occupying over 60% of the arterial tree.^47^ In this study, we observed very high serum LDL-C levels but did not observe any plaque formation as the study was executed in mice at the average age of 11 weeks. When DKO mice are fed the Western-type diet, they rapidly develop atherosclerosis at a very early age.^46^ Our group has previously shown that the gene transfer of AAV8 vector expressing a murine form of LDLR reduced plasma cholesterol and showed complete regression of atherosclerosis (aortic plaque formation) despite the continuation of a Western-type diet throughout the study.^48^ The current study focused on developing second-generation human LDLR vectors to achieve therapeutic effects at a low dose. Future studies could be conducted to examine whether AAV8-IVS2.hLDLR011-T and AAV8-IVS2.hLDLR011 effectively reduce atherosclerotic lesion progression in DKO mice fed a Western diet. The advantages of the LDLR triple variant in IVS2.hLDLR011-T can also be confirmed by performing studies in DKO mice overexpressing PCSK9 and/or IDOL.

To treat HoFH, long-term gene transfer is required in patients to effectively control hypercholesterolemia and atherosclerotic plaque formation. We have previously shown that the hepatocyte-targeted AAV8 vector with the liver-specific TBG promoter induces persistent and stable transgene expression for up to 180 days in DKO mice.^29^ In the current study, serum LDL levels remained as low as 1% of pre-treatment levels in DKO mice for 120 days following a single injection of 7.5 × 10^12^ GC/kg AAV8-IVS2.hLDLR011-T. Persistent transgene expression of AAV8-IVS2.hLDLR011-T was observed for 120 days in this study, although vector DNA and transgene expression levels reduced by ~4-fold compared with those in the peak time. Reduction of vector genome copies and/or transgene gene expression levels during the later phase compared with the early peak time has been observed in liver-directed gene therapy studies in animal models and in clinical trials,^10^^,^^13^^,^^26^^,^^49^ which could be potentially attributed to the AAV vector biology, turnover of the transduced hepatocytes over time, or mechanisms related to host immune response. A long-term dose-response study in DKO mice can also be performed to investigate the role played by overexpression of LDLR. The findings presented here demonstrate that the combination of codon optimization and incorporation of IVS2 or WPRE enhanced transgene expression and reduced LDL-C levels more efficiently than the first generation AAV8-PI.hLDLR-WT vector. Based on our results, the second-generation clinical candidate vector we have developed, AAV8-IVS2.hLDLR011-T, is a promising vector to achieve therapeutic effects in HoFH patients.

## Materials and Methods

### Codon optimization and vector construction and production

In order to improve the expression of h*LDLR*, we inserted a Kozak sequence (GCCACC) before the start codon of the original AAV construct containing the native h*LDLR* cDNA (referred to as hLDLR-WT).^50^ Codon optimization was performed as described in the patent application WO2015012924. Specifically, codon bias tables B, D, and E from WO2015012924 were used to reverse-translate the amino acid sequence of LDLR. In addition, a separate codon bias table was created based on the native sequence of the macaque antibody IgG1-201^51^ and applied to LDLR amino acid sequence to generate a new coding sequence. Four different codon-optimized cDNA sequences were synthesized and cloned into the AAV plasmids. GeneScript Rare Codon Analysis Tool (https://www.genscript.com/tools/rare-codon-analysis) was used to evaluate the properties of the hLDLR-WT and optimized h*LDLR* cDNA that include CAI, guanine-cytosine content (GC-content), and codon frequency distribution. Further vector optimization was performed on h*LDLR*011 by replacing the PI intron with IVS2 (Data S1) from the human beta-globin gene. h*LDLR*011-T was derived from hLDLR011 by site-directed mutagenesis using QuickChange Lightning kit (Agilent, Santa Clara, CA, USA) to incorporate the *LDLR* triple-mutant variant (hLDLR-L318D/K809R/C818A) to the hLDLR011 cDNA sequence (referred to as hLDLR011-T). WPRE (Data S1) was inserted at the 3′ end of the hLDLR-T cDNA. All AAV vectors were produced by triple transfection in 293 cells and the Penn Vector Core at the University of Pennsylvania as described previously.^52^^,^^53^

### Animal studies

All animal studies were performed in accordance with protocols approved by Institutional Animal Care and Use Committee (IACUC) of the University of Pennsylvania. *Ldlr*^−/−^, *Apobec1*^−/−^ DKO mice were maintained on a chow diet at an animal facility in the University of Pennsylvania. DKO mice, aged 9–15 weeks (mean age, 11 weeks), were enrolled into the study. Vectors were injected intravenously via the tail vein at the specified dose. Blood was collected before vector dosing and at specified time points after dosing.

### Serum non-HDL-C or LDL-C levels

Serum non-HDL-C levels were measured as previously described.^33^ Serum LDL-C levels were measured by Antech GLP (Morrisville, NC, USA). Data after vector treatment are also presented as percentage of baseline levels.

### Quantification of vector genomes and transgene mRNA in liver

Vector genomes in liver were quantified by qPCR (TaqMan Universal MasterMix; Applied Biosystems/Thermo Fisher Scientific, Waltham, MA, USA) as described previously.^54^ RNA isolation and qRT-PCR were performed as described previously.^55^

### IHC staining and ISH

IHC staining to detect hLDLR protein was performed as previously described.^48^ ISH for hLDLR011 was performed as previously described using Z-shaped probe pairs binding to hLDLR011.^55^

### Western blot analysis

Western analysis on proteins in liver lysate (20 μg/lane) was performed as previously described.^25^ LDLR and beta-tubulin were detected with rabbit anti-LDLR antibody (ab52818; Abcam, Cambridge, MA, USA) and rabbit anti-beta-tubulin antibody (ab6046; Abcam), respectively. Bound primary antibody was detected with horseradish peroxidase-conjugated goat anti-rabbit IgG antibody (Thermo Fisher) and SuperSignal West Pico Chemiluminescence Substrate (Thermo Fisher).

### Statistical analyses

GraphPad Prism 9.0 was used for statistical analyses. An unpaired Student’s t test was used for comparison of vector genome copies between females and males. Comparisons between multiple groups or multiple time points were performed using one-way analysis of variance (ANOVA) followed by Dunnett’s multiple comparison test or Tukey’s multiple comparison test. All values are expressed as mean ± SEM.

### Data availability statement

All data and supporting materials are available within the article and Supplemental information.

## Supplemental information

Document S1. Tables S1 and S2, Figure S1, and Data S1

Document S2. Article plus supplemental information
