# Supplementary material for: Developing a second-generation clinical candidate AAV vector for gene therapy of familial hypercholesterolemia
Source: Mol Ther Methods Clin Dev. 2021 May 5;22:1–10. doi: 10.1016/j.omtm.2021.04.017 (PMC8237527; doi:10.1016/j.omtm.2021.04.017)
Supplement: Document S1. Tables S1 and S2, Figure S1, and Data S1 [file mmc1.pdf]

OMTM, Volume *22*

Supplemental information

Developing a second-generation clinical candidate AAV vector for gene therapy of familial hypercholesterolemia

Lili Wang, Ilayaraja Muthuramu, Suryanarayan Somanathan, Hong Zhang, Peter Bell, Zhenning He, Hongwei Yu, Yanqing Zhu, Anna P. Tretiakova, and James M. Wilson

Table S1. Significance of reduction of serum LDL-C levels in female and male DKO mice on days 14 and 28 after administration of AAV8.hLDLR vectors at different doses.

Vector	F, $3x10^{11}$ GC/kg		F, $1x10^{12}$ GC/kg		M, $1x10^{11}$ GC/kg		M, $3x10^{11}$ GC/kg		M, $1x10^{12}$ GC/kg	
(n=5)	d14	d28	d14	d28	d14	d28	d14	d28	d14	d28
PI.hLDLR-WT	ns	(**)	**	*	****	***	*	**	****	****
PI.hLDLR011	ns	ns	**	**	**	***	****	****	****	****
IVS2.hLDLR011	ns	ns	***	***	****	****	***	***	***	***
IVS2.hLDLR011-T	ns	ns	***	***	****	***	****	****	****	****
PI.hLDLR011-T +WPRE	ns	ns	***	**	****	****	****	****	****	****

Serum LDL-C levels on days -1 (d-1), 14 (d14), and 28 (d28) are shown in Figure 2C. GraphPad Prism 9.0 was used for statistical analyses. For comparisons of LDL-C before (d-1) and after vector treatment (d14 and d28), RM one-way ANOVA with Geisser-Greenhouse correction was applied, with Dunnett's multiple comparison test to test for significant differences on d14 and d28 after vector treatment. * $p<0.05$, ** $p<0.01$, *** $p<0.001$, ****$p<0.0001$. (**) indicates d28 levels were significantly higher than d-1 levels.

Table S2. Comparison of the level of serum LDL-C reduction in female and male DKO mice treated with different AAV8.hLDLR vectors.

Day 14	Vector	PBS	PI.hLDLR-WT	PI.hLDLR011	IVS2.hLDLR011	IVS2.hLDLR011-T
Female DKO	PBS					
	PI.hLDLR-WT	ns				
	PI.hLDLR011	ns	ns			
$3x10^{11}$ GC/kg	IVS2.hLDLR011	*	*	ns		
	IVS2.hLDLR011-T	ns	ns	ns	ns	
	PI.hLDLR011-T+WPRE	ns	ns	ns	ns	ns

Day 28	Vector	PBS	PI.hLDLR-WT	PI.hLDLR011	IVS2.hLDLR011	IVS2.hLDLR011-T
Female DKO	PBS					
	PI.hLDLR-WT	ns				
	PI.hLDLR011	ns	ns			
$3x10^{11}$ GC/kg	IVS2.hLDLR011	***	****	*		
	IVS2.hLDLR011-T	*	**	ns	ns	
	PI.hLDLR011-T+WPRE	ns	ns	ns	**	ns

Day 14	Vector	PBS	PI.hLDLR-WT	PI.hLDLR011	IVS2.hLDLR011	IVS2.hLDLR011-T
Female DKO	PBS					
	PI.hLDLR-WT	****				
	PI.hLDLR011	****	ns			
$1x10^{12}$ GC/kg	IVS2.hLDLR011	****	***	ns		
	IVS2.hLDLR011-T	****	**	ns	ns	
	PI.hLDLR011-T+WPRE	****	**	ns	ns	ns

Day 28	Vector	PBS	PI.hLDLR-WT	PI.hLDLR011	IVS2.hLDLR011	IVS2.hLDLR011-T
Female DKO	PBS					
	PI.hLDLR-WT	***				
	PI.hLDLR011	****	ns			
$1x10^{12}$ GC/kg	IVS2.hLDLR011	****	***	*		
	IVS2.hLDLR011-T	****	***	ns	ns	
	PI.hLDLR011-T+WPRE	****	*	ns	ns	ns

Day 14	Vector	PBS	PI.hLDLR-WT	PI.hLDLR011	IVS2.hLDLR011	IVS2.hLDLR011-T
Male DKO	PBS					
	PI.hLDLR-WT	***				
	PI.hLDLR011	****	****			
$1x10^{11}$ GC/kg	IVS2.hLDLR011	****	****	****		
	IVS2.hLDLR011-T	****	****	****	ns	
	PI.hLDLR011-T+WPRE	****	****	****	ns	ns

Day 28	Vector	PBS	PI.hLDLR-WT	PI.hLDLR011	IVS2.hLDLR011	IVS2.hLDLR011-T
Male DKO	PBS					
	PI.hLDLR-WT	****				
	PI.hLDLR011	****	**			
$1x10^{11}$ GC/kg	IVS2.hLDLR011	****	****	****		
	IVS2.hLDLR011-T	****	****	**	ns	
	PI.hLDLR011-T+WPRE	****	****	****	ns	ns

Day 14	Vector	PBS	PI.hLDLR-WT	PI.hLDLR011	IVS2.hLDLR011	IVS2.hLDLR011-T

Male DKO	PBS					
	PI.hLDLR-WT	****				
3x10^{11} GC/kg or 1x10^{12} GC/kg	PI.hLDLR011	****	****			
	IVS2.hLDLR011	****	****	ns		
	IVS2.hLDLR011-T	****	****	ns	ns	
	PI.hLDLR011-T+WPRE	****	****	ns	ns	ns

Day 28	Vector					
Male DKO	PBS					
	PI.hLDLR-WT	****				
3x10^{11} GC/kg or 1x10^{12} GC/kg	PI.hLDLR011	****	****			
	IVS2.hLDLR011	****	****	ns		
	IVS2.hLDLR011-T	****	****	ns	ns	
	PI.hLDLR011-T+WPRE	****	****	ns	ns	ns

Serum LDL-C levels on days 14 and 28 were normalized by baseline levels on day -1 and are shown in Figure 2D. GraphPad Prism 9.0 was used for statistical analyses. For comparisons of efficiency of different vectors at the specified dose, time point and sex of mice, one-way ANOVA was applied followed by Tukey’s multiple comparison test. * $p<0.05$, ** $p<0.01$, *** $p<0.001$, ****$p<0.0001$. ns, not significant.

A

	GC content (%)	CAI	Rare codon percentage
hLDLR-WT	58.38	0.83	1
hLDLR-kozak	58.41	0.83	1
hLDLR001	67.02	0.97	0
hLDLR011	60.70	0.95	0
hLDLR201	62.66	0.96	0
hLDLR026	62.16	0.91	0

B

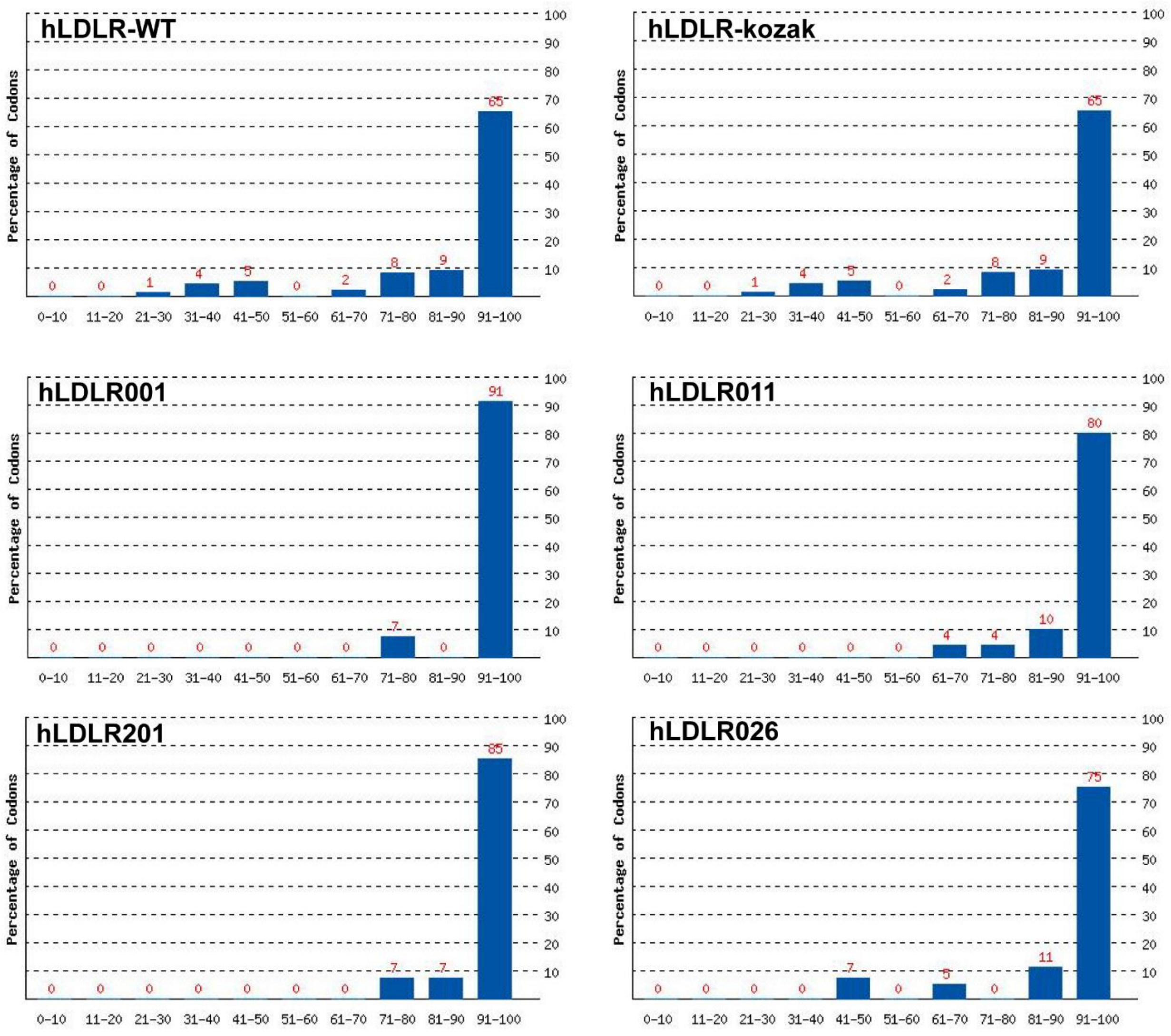

Figure S1. Rare codon analysis on the hLDLR cDNA sequences by GeneScript Rare Codon Analysis Tool. (A) GC content, CAI score, and rare codon percentage in the hLDLR cDNA sequences. (B) Analysis of codon frequency distribution. The value of 100 is set for the codon with highest usage frequency for a given amino acid in the host expression organism (human). Codons with values lower than 30 are considered to hinder protein expression.

Data file S1: Sequences for IVS2 and WPRE.

FASTA sequences:

>IVS2 572 bp

AGCTTACTTGTGGTACCGAGCTCGGATCCTGAGAACTTCAGGGTGAGTCTATGGGACCCTTG
ATGTTTTCTTTCCCCTTCTTTTCTATGGTTAAGTTCATGTCATAGGAAGGGGAGAAGTAACAG
GGTACACATATTGACCAAATCAGGGTAATTTTGCATTTGTAATTTTAAAAAATGCTTTCTTCT
TTTAATATACTTTTTTGTTTATCTTATTTCTAATACTTTCCCTAATCTCTTTCTTTCAGGGCAAT
AATGATACAATGTATCATGCCTCTTTGCACCATTCTAAAGAATAACAGTGATAATTTCTGGG
TTAAGGCAATAGCAATATTTCTGCATATAAATATTTCTGCATATAAATTGTAACTGATGTAA
GAGGTTTCATATTGCTAATAGCAGCTACAATCCAGCTACCATTCTGCTTTTATTTTATGGTTG
GGATAAGGCTGGATTATTCTGAGTCCAAGCTAGGCCCTTTTGCTAATCATGTTCATACCTCTT
ATCTTCCTCCCACAGCTCCTGGGCAACGTGCTGGTCTGTGTGCTGGCCCATCACTTTGGCAAA
GAATTG

>WPRE 542 bp

AATCAACCTCTGGATTACAAAATTTGTGAAAGATTGACTGGTATTCTTAACTATGTTGCTCCT
TTTACGCTATGTGGATACGCTGCTTTAATGCCTTTGTATCATGCTATTGCTTCCCGTATGGCTT
TCATTTTCTCCTCCTTGTATAAATCCTGGTTGCTGTCTCTTTATGAGGAGTTGTGGCCCGTTGT
CAGGCAACGTGGCGTGGTGTGCACTGTGTTTGCTGACGCAACCCCCACTGGTTGGGGCATTG
CCACCACCTGTCAGCTCCTTTCCGGGACTTTCGCTTTCCCCCTCCCTATTGCCACGGCGGAAC
TCATCGCCGCCTGCCTTGCCCGCTGCTGGACAGGGGCTCGGCTGTTGGGCACTGACAATTCC
GTGGTGTTGTCGGGGAAATCATCGTCCTTTCCTTGGCTGCTCGCCTGTGTTGCCACCTGGATT
CTGCGCGGGACGTCCTTCTGCTACGTCCCTTCGGCCCTCAATCCAGCGGACCTTCCTTCCCGC
GGCCTGCTGCCGGCTCTGCGGCCTCTTCCGCGTCTTCG
