# Supplementary material for: Developing a second-generation clinical candidate AAV vector for gene therapy of familial hypercholesterolemia
Source: Mol Ther Methods Clin Dev. 2021 May 5;22:1–10. doi: 10.1016/j.omtm.2021.04.017 (PMC8237527; doi:10.1016/j.omtm.2021.04.017)
Supplement: Document S2. Article plus supplemental information [file mmc2.pdf]

Molecular Therapy
Methods & Clinical Development
Original Article

Developing a second-generation clinical candidate AAV vector for gene therapy of familial hypercholesterolemia

Lili Wang,[1] Ilayaraja Muthuramu,[1] Suryanarayan Somanathan,[1,2] Hong Zhang,[1] Peter Bell,[1] Zhenning He,[1] Hongwei Yu,[1] Yanqing Zhu,[1] Anna P. Tretiakova,[1,3] and James M. Wilson[1]

[1]Gene Therapy Program, Department of Medicine, Perelman School of Medicine, University of Pennsylvania, Philadelphia, PA 19104, USA

Gene therapy for hypercholesterolemia offers the potential to sustainably ameliorate disease for life with a single dose. In this study, we demonstrate the combinatorial effects of codon and vector optimization, which significantly improve the efficacy of an adeno-associated virus (AAV) vector in the low-density lipoprotein receptor (LDLR)-deficient mouse model (*Ldlr*$^{-/-}$, *Apobec1*$^{-/-}$ double knockout [DKO]). This study investigated vector efficacy following the combination of intervening sequence 2 (IVS2) of the human beta-globin gene and codon optimization with the previously developed gain-of-function, human LDLR triple-mutant variant (hLDLR-L318D/K809R/C818A) in the treatment of homozygous familial hypercholesterolemia (HoFH). Vector doses as low as 3×10^{11} genome copies (GC)/kg achieved a robust reduction of serum low-density lipoprotein cholesterol (LDL-C) by 98% in male LDLR-deficient mice. Less efficient LDL-C reduction was observed in female mice, which was attributable to lower gene transfer efficiency in liver. We also observed persistent and stable transgene expression for 120 days, with LDL-C levels being undetectable in male DKO mice treated with the second-generation vector. In conclusion, codon and vector optimization enhanced transgene expression and reduced serum LDL-C levels effectively at a lower dose in LDLR-deficient mice. The second-generation clinical candidate vector we have developed has the potential to achieve therapeutic effects in HoFH patients.

INTRODUCTION

Homozygous familial hypercholesterolemia (HoFH) is an autosomal-dominant monogenic disease that affects more than 30 million people worldwide.[1] HoFH is primarily caused by mutations in three genes: low-density lipoprotein receptor (*LDLR*), apolipoprotein B100 (*APOB-100*), and proprotein convertase subtilisin/kexin type 9 (*PCSK9*).[2] More than 1,200 molecular defects have been identified in the *LDLR* gene, with such mutations significantly impairing or ablating the receptor's proper functionality. Homozygous patients with mutations affecting both alleles of the *LDLR* gene have very high low-density lipoprotein cholesterol (LDL-C) concentrations, exhibit premature coronary atherosclerosis, and develop cardiovascular disease before 30 years of age. Heterozygous familial hypercholesterolemia (HeFH) patients with one abnormal *LDLR* allele have clinical evidence of coronary artery disease (CAD) by 45 years of age.[3]

For most HoFH patients, the current lipid-lowering therapies, such as plasmapheresis, LDL apheresis, or liver transplantation, and cholesterol-lowering drugs (e.g., high-dose statins) are not able to reduce LDL-C to normal levels and also have adverse side effects.[4–6] In recent years, PCSK9 inhibitors, such as evolocumab and alirocumab, have emerged as the newer generation of lipid-lowering drugs and can significantly reduce LDL-C in HeFH and non-familial hypercholesterolemia (non-FH) patients.[7] However, the results have been variable in HoFH patients,[8] and new therapeutic strategies are being sought. LDLR activity plays a vital role in mediating PCSK9 inhibitor efficacy, such that HoFH patients with a bi-allelic mutant *LDLR* gene or patients with residual LDLR activity <2% are considered receptor-negative subjects who lack any responses to PCSK9 inhibitor treatment.[8] Gene therapy provides the possibility of stable and long-term expression of LDLR to correct the fundamental defect in HoFH.[9] In recent years, liver-directed gene therapy by adeno-associated virus (AAV) vectors has shown encouraging efficacy in clinical trials for hemophilia A or B patients in which sustained therapeutic expression of clotting factor VIII or IX for up to 3 years was reported.[10–13] Based on efficacy data in preclinical studies in *LDLR*-deficient mouse models and pharmacological studies in nonhuman primates,[14,15] the first-in-human clinical trial using AAV8.hLDLR for treatment in HoFH was started in 2016.[16] However, one subject in cohort 1 [2.5×10^{12} genome copies (GC)/kg] and all three subjects in cohort 2 [7.5×10^{12} GC/kg] experienced an asymptomatic elevation in transaminases 4–6 weeks post-dosing and had positive T cell response to the AAV capsid.[17] Efficacy data have not yet been reported.

Received 10 February 2021; accepted 29 April 2021;
https://doi.org/10.1016/j.omtm.2021.04.017.

[2]Present address: Pfizer, Inc., Cambridge, MA, USA
[3]Present address: AskBio, Research Triangle Park, NC, USA
Correspondence: James M. Wilson, MD, PhD, Gene Therapy Program, Department of Medicine, Perelman School of Medicine, University of Pennsylvania, 125 South 31st Street, Suite 1200, Philadelphia, PA 19104, USA.
E-mail: wilsonjm@upenn.edu

This is an open access article under the CC BY license (http://creativecommons.org/licenses/by/4.0/).

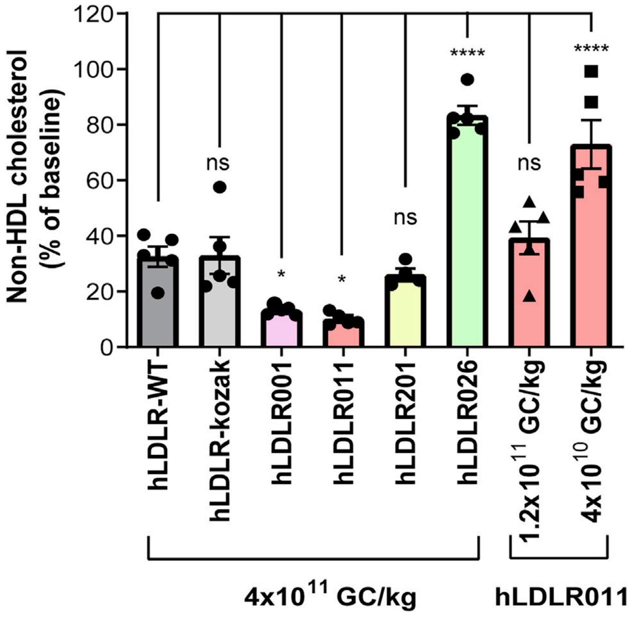

Figure 1. Evaluation of different codon-optimized hLDLR cDNA sequences in male DKO mice

Serum non-HDL cholesterol levels in male DKO mice were reduced 14 days after intravenous administration of AAV8.hLDLR vectors. Serum non-HDL levels are shown as percentage of baseline levels. All six vectors were tested at 4×10^{11} GC/kg. LDLR011 was also evaluated at 1.2×10^{11} and 4×10^{10} GC/kg. Individual mouse data (five mice per group) and means ± SEM are shown. Each group was compared to the hLDLR-WT group. *$p < 0.05$, ****$p < 0.0001$, one-way ANOVA followed by Dunnett's multiple comparison test.

The liver plays a crucial role in cholesterol homeostasis by regulating LDLR expression via a negative feedback mechanism that controls plasma cholesterol and maintains intracellular cholesterol levels.[18] Hepatic *LDLR* is tightly regulated at the transcriptional level by sterol-regulatory element-binding protein (SREBPs), and at the post-transcriptional level by the PCSK9 and inducible degrader of LDLR (IDOL) pathways.[19] SREBPs control the regulation of both *PCSK9* and *LDLR* expression, which primarily mediate the effects of statin treatment. Statins reduce LDL-C indirectly by increasing *LDLR* expression, but this effect is diminished by increased *PCSK9* expression.[20,21] Based on human genetics studies and molecular mapping, the PCSK9-LDLR binding interface plays a crucial role in regulating circulating levels of LDL-C. PCSK9 has thus become a therapeutic target, with numerous clinical trials showing that PCSK9 inhibitors can safely reduce LDL-C levels up to 70%.[22,23]

We previously reported the development and evaluation of an AAV8 vector expressing a gain-of-function, human *LDLR* triple-mutant variant (hLDLR-L318D/K809R/C818A) in which this LDLR variant exhibited partial resistance to degradation via PCSK9 and IDOL pathways in mouse models of *LDLR* deficiency.[24] The partial resistance to degradation in this gain-of-function LDLR variant was found to be dose dependent in the presence of human PCSK9 or IDOL overexpression.[24] In this study, we aimed to develop a more efficient second-generation AAV vector for gene therapy of HoFH through codon and vector optimization. We report that the combination of codon and vector construct optimization increased the efficacy of the AAV.hLDLR vector by 10-fold. The combination of codon and vector optimization, the triple-mutant LDLR variant, and an efficient liver-tropic AAV capsid suggests that this vector has great potential to safely achieve clinical benefits in HoFH patients at a low dose.

RESULTS

Improve LDLR expression by codon optimization

To improve expression levels of human *LDLR* in the AAV vector, we first incorporated the Kozak sequence in front of the start codon; the native h*LDLR* sequence (wild type [WT]) does not contain the Kozak consensus sequence, which has been shown to enhance translation from the correct initiation codon.[25] When comparing the two vectors in *Ldlr*$^{-/-}$, *Apobec1*$^{-/-}$ double-knockout (DKO) mice, however, the two groups of mice showed similar levels of non-HDL-C reduction 14 days after vector treatment (Figure 1). Because codon optimization has been shown to increase transgene expression levels in AAV vectors,[26] we designed four codon-optimized h*LDLR* cDNA sequences that also contain the Kozak consensus sequence to replace the WT h*LDLR* cDNA sequence in the AAV vector and packaged each construct in AAV8 capsids for *in vivo* evaluation in male DKO mice. Some properties of the cDNA, such as codon adaptation index (CAI), guanine-cytosine (GC)-content, rare codon percentage, and codon frequency distribution, are shown in Figure S1.

To determine which codon-optimized hLDLR reduced serum non-HDL-C more efficiently, we performed a pilot study that involved injecting 4×10^{11} GC/kg AAV8-hLDLR vectors intravenously in male DKO mice. Fourteen days after vector treatment, serum non-HDL-C levels reduced: AAV8.hLDLR-WT by 68%, hLDLR-Kozak by 67%, hLDLR001 by 87%, hLDLR011 by 90%, hLDLR201 by 74%, and hLDLR026 by 17% (Figure 1). The hLDLR026 variant showed significantly less efficient reduction compared with hLDLR-WT, thereby indicating that not all codon optimization necessarily leads to improved expression. We further evaluated the dose effect of hLDLR011 at two lower doses, 1.2×10^{11} and 4×10^{10} GC/kg. hLDLR011 did not show efficient reduction at 4×10^{10} GC/kg. At 1.2×10^{11} GC/kg, hLDLR011 showed 61% reduction of LDL-C, similar to hLDLR-WT at 4×10^{11} GC/kg (Figure 1), suggesting hLDLR011 is about 3-fold more efficient than hLDLR-WT. Based on the pilot study data, we proceeded with hLDLR011 as the cDNA sequence for further vector optimization. In addition, we introduced the previously demonstrated gain-of-function triple mutations (hLDLR-L318D/K809R/C818A) to confer partial resistance to LDLR degradation via the PCSK9 or IDOL pathway to the hLDLR011 cDNA sequence to generate hLDLR011-T.[24]

Improve LDLR expression by vector optimization

To further improve h*LDLR* expression from AAV vectors containing the liver-specific thyroxine-binding globulin (TBG) promoter, we evaluated the effects of a different intron sequence, intervening sequence 2 (IVS2) of the human beta-globin gene or the WPRE

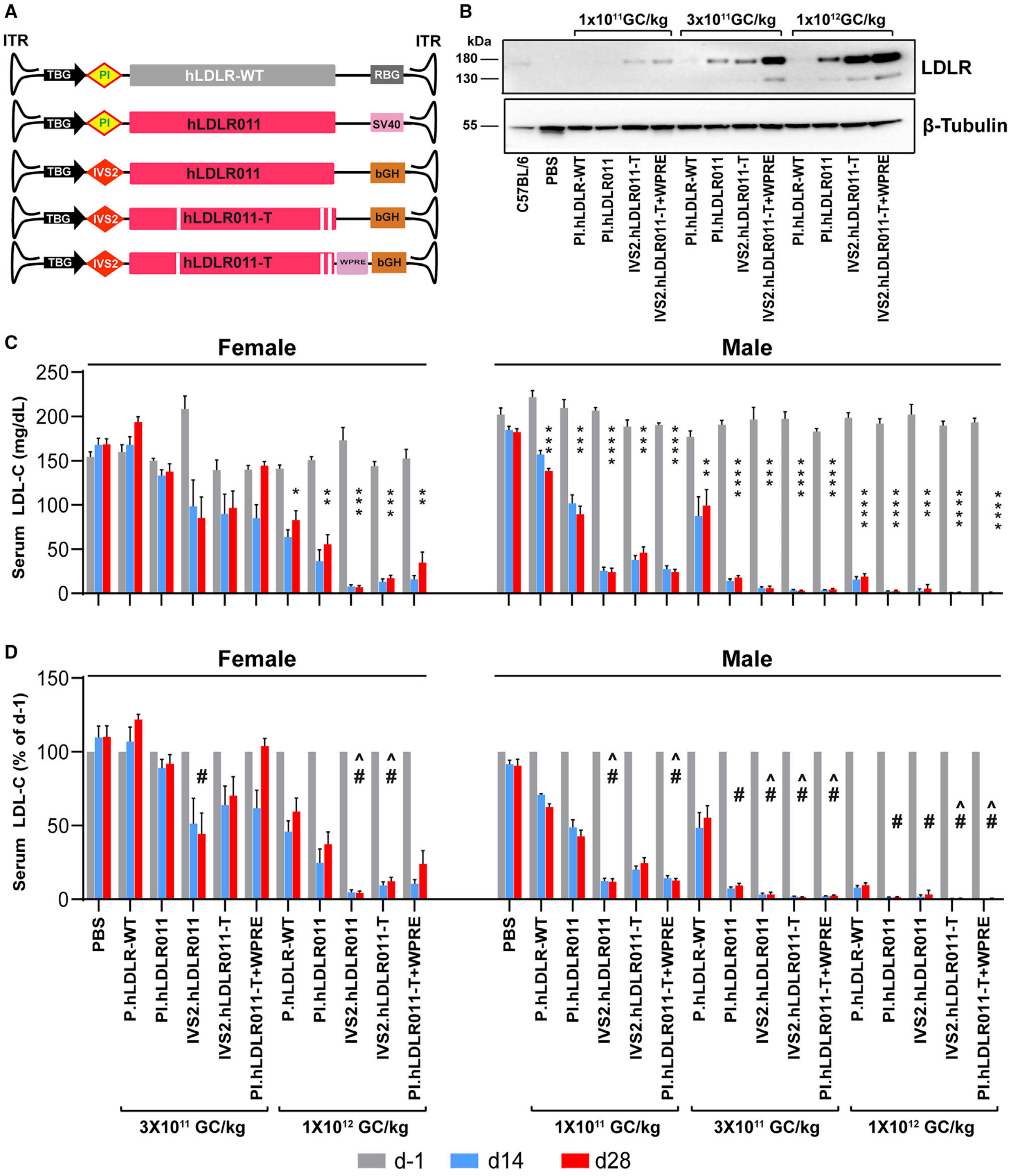

Figure 2. Vector optimizations improve efficacy in DKO mice

(A) Schematic illustration of different AAV.hLDLR vector constructs. (B) Western blot analysis on liver lysates (20 μg/lane) from male DKO mice 28 days post-administration of PBS or AAV8.hLDLR vectors at 1×10^{11}, 3×10^{11}, and 1×10^{12} GC/kg. Liver lysate from an untreated C57BL/6 mouse was included as a reference. One representative sample from each group is shown. (C) Serum LDL-C levels at 1 day before (d−1) or 14 and 28 days (d14 and d28, respectively) after administration of PBS or AAV8.hLDLR vectors at the indicated doses in female and male DKO mice expressed as mg/dL. The significance of reduction on d28 compared with d−1 is indicated. Mean ± SEM are

(legend continued on next page)

(woodchuck hepatitis virus post-transcriptional regulatory element) at the 3′ end of the cDNA in the expression cassette (Figure 2A). These two elements have been previously used in AAV vectors for hemophilia B gene therapy and achieved efficient clotting factor IX expression.[27,28] We then compared the efficacy of the hLDLR-WT and four of the optimized hLDLR011 vectors in both female and male DKO mice at multiple vector doses. Liver tissues were harvested from male DKO mice on day (d) 28 post-vector administration for Western blot analyses to evaluate hLDLR protein levels in liver lysate. Vector- and dose-dependent effects on LDLR protein expression were observed. PI.hLDLR-WT (vector currently being used in the clinical trial ClinicalTrials.gov: NCT02651675) hardly showed any detectable signals even at the highest dose tested (1×10^{12} GC/kg), while hLDLR011 vectors containing IVS2 or WPRE showed LDLR signals at the lowest dose tested (1×10^{11} GC/kg) (Figure 2B). We measured serum LDL-C levels in mice 1 day before dosing (d−1), as well as 14 and 28 days after vector dosing, and levels are shown in Figure 2C. In female DKO mice, none of the 3×10^{11} GC/kg dose groups showed significant reduction of serum LDL-C at d14 or d28 after dosing (Figure 2C; Table S1). However, when comparing normalized LDL-C levels (percentage of d−1) between different vectors at the 3×10^{11} GC/kg dose in females, the two IVS2-containing vectors performed significantly better than the other groups (Figure 2D; Table S2). At 1×10^{12} GC/kg, all vectors significantly reduced serum LDL-C levels in females (Figure 2C; Table S1), with the two IVS2-containing vectors showing more significant reduction than the other three vectors (Figure 2D; Table S2). Male DKO mice showed more robust reduction compared with female mice, consistent with our previous observations in DKO mice using the murine *LDLR* or human *LDLR* cDNA.[14] Male mice treated with hLDLR011 vectors at 3×10^{11} GC/kg showed 93%–98% reduction of LDL-C on d14 after vector treatment, while the hLDLR-WT vector-treated mice showed 52% of reduction (Figure 2D). At 1×10^{12} GC/kg dose, LDL-C levels in hLDLR011 vector-treated male mice were almost undetectable. We therefore treated the males with a lower dose (1×10^{11} GC/kg) and found that vectors containing IVS2 or WPRE showed enhanced LDL-C reduction compared with PI.h*LDLR*011 alone (Figure 2D; Table S2). Immunohistochemical (IHC) staining of hLDLR in liver harvested at d28 after vector treatment showed strong staining on the lining of most hepatocytes in male DKO mice treated with hLDLR011 or hLDLR011-T vectors (Figure 3B) compared with the fewer positive hepatocytes in female DKO mice (Figure 3A).

More efficient gene transfer in the liver of male DKO mice than in female DKO mice

In one of our previous studies to evaluate AAV8.TBG.PI.hLDLR-WT vector biodistribution in DKO mice at the dose of 7.5×10^{12} GC/kg, male mice showed slightly higher vector genome copies in liver than the female mice. However, these differences were only significant at a later time point of d180, but not on d3, d14, and d90.[29] To determine whether gene transfer efficiency was affected by sex in the DKO mice in this study when vector doses were 7.5- to 25-fold lower, we measured vector genome copies in d28 liver samples from male and female mice treated with IVS2.h*LDLR*011-T at two vector doses (3×10^{11} and 1×10^{12} GC/kg). Male mice had significantly higher (3- to 5-fold higher) vector genome copies in the liver than the female mice at both doses (Figure 4). Males treated with 3×10^{11} GC/kg had similar vector genome copies as the female mice treated with 1×10^{12} GC/kg, suggesting AAV8 gene transfer was about 3-fold higher in male DKO mice than in female mice. Mice with vector genome copies in liver as low as 0.2 copies per diploid genome (female at 1×10^{12} GC/kg and male at 3×10^{11} GC/kg, respectively) demonstrated efficient reduction of LDL-C at 90%–98% (Figures 2B and 2C).

Enhancing h*LDLR* mRNA levels by IVS2 and WPRE

To confirm the roles of IVS2 and WPRE in enhancing h*LDLR* mRNA levels, we compared the h*LDLR* mRNA levels in the liver of male DKO mice treated with 1×10^{12} GC/kg hLDLR vectors. IVS2-containing vectors showed 5.7-, 3.6-, and 1.6-fold higher *LDLR* mRNA levels than Promega intron (PI)-hLDLR-WT, PI.hLDLR011, and PI.hLDLR011-T + WPRE, respectively (Figure 5A). The WPRE-containing vector showed 3.6- and 2.4-fold higher *LDLR* mRNA levels than h*LDLR*-WT and PI.hLDLR011, respectively (Figure 5A); these data are consistent with previous reports on the function of WPRE.[30–32] Vector genome copy analysis by quantitative PCR (qPCR) showed no statistical differences between the mice treated with different vectors (Figure 5B), indicating equivalent gene transfer efficiency by the four vectors, and the increased mRNA levels were attributed by IVS2 or WPRE. The size limitation of the AAV vector does not allow the h*LDLR* vector to contain both IVS2 and WPRE; we have previously reported the development and evaluation of gain-of function, human *LDLR* triple-mutant variant (hLDLR-L318D/K809R/C818A), which exhibits partial resistance to degradation via PCSK9 and IDOL pathways.[24] Therefore, we selected IVS2.hLDLR011-T for further evaluation.

Kinetics of LDLR expression in male DKO mice

To evaluate the kinetics of the IVS2.hLDLR011-T vector, we dosed male DKO mice with AAV8.IVS2.hLDLR011-T vector at 7.5×10^{12} GC/kg, the same dose being administered to cohort 2 in the current AAV8 gene therapy trial for HoFH.[16] One day after dosing, LDL-C levels were quickly reduced by 77% and reached a barely detectable level by d3 that persisted for the remainder of the study duration (d120) (Figure 6A). The robustness of LDL-C reduction was not

shown (n = 5/group). *p < 0.05, **p < 0.01, ***p < 0.001, ****p < 0.0001. One-way ANOVA followed by Dunnett's multiple comparison test. Detailed statistical differences after treatment on d14 and d28 are shown in Table S1. (D) Serum LDL-C levels shown as percentage of levels at d−1. Means ± SEM are shown (n = 5/group). Vectors with d28 serum LDL-C (% of d−1) levels significantly lower than the levels in PI.hLDLR-WT (#) or PI.hLDLR011 (^), respectively, within the same dose and sex cohorts. One-way ANOVA followed by Tukey's multiple comparison test. Detailed statistical differences between different vectors on d14 and d28 are shown in Table S2. bGH, bovine growth hormone polyA; hLDLR011-T, LDLR011 carrying the triple-mutant variant (hLDLR-L318D/K809R/C818A); IVS2, intervening sequence 2 of human beta-globin gene; PI, Promega intron; RBG, rabbit beta-globulin polyA; SV40, SV40 polyA; WPRE, woodchuck hepatitis virus post-transcriptional regulatory element.

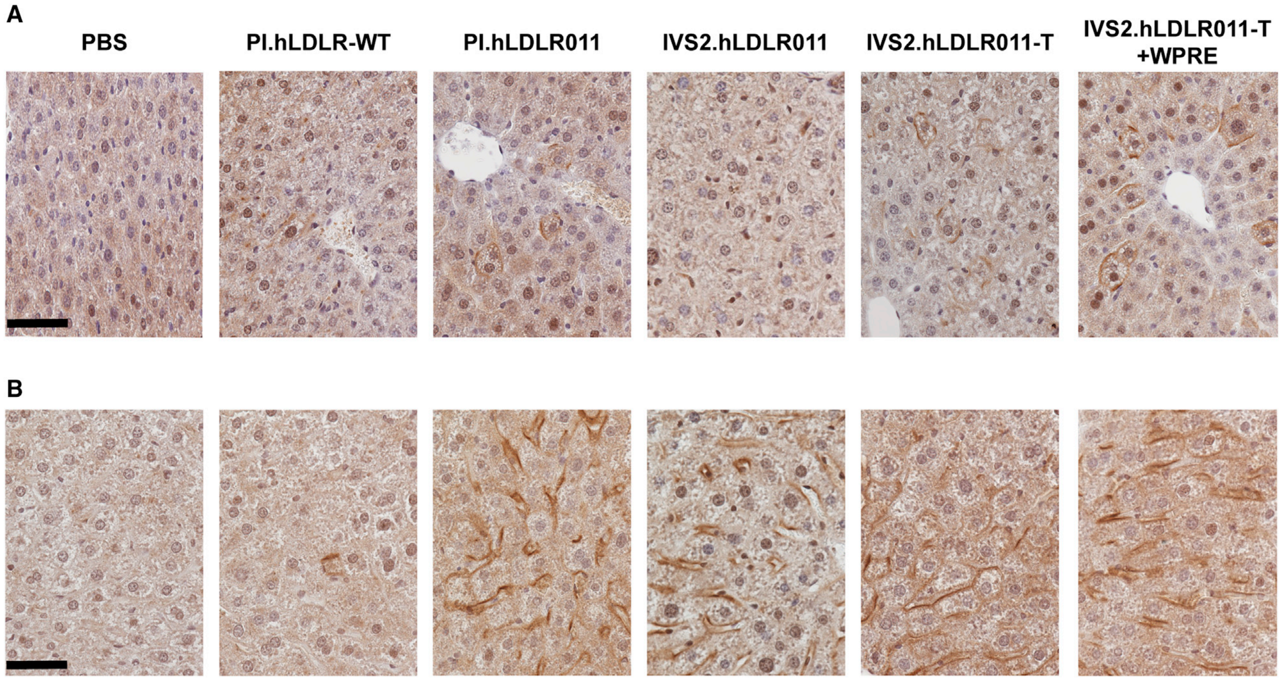

Figure 3. Vector optimizations improve LDLR protein expression in DKO mice

(A and B) Livers were harvested 28 days after AAV8.hLDLR vector administration at 3×10^{11} GC/kg in female (A) and male (B) DKO mice for immunohistochemical staining for hLDLR. Representative images are shown. Scale bar, 50 μm.

achieved by PI.hLDLR-WT, the first-generation FH gene therapy vector.[14,33] We harvested liver tissue (n = 5 mice/time point) on d1, d3, d7, d14, and d120 after vector administration. Vector genome copies in the liver measured by qPCR were high at d1 post-dosing, but then declined over time (Figure 6B). We detected a 4-fold reduction of vector genome in liver between d14 and d120. On the other hand, h*LDLR* mRNA expression increased from d1 to d14 before decreasing by 3.6-fold on d120, a rate similar to the vector DNA reduction (Figure 6B). Liver samples collected at each time point were also examined by IHC staining and *in situ* hybridization (ISH) for detection of LDLR protein and h*LDLR* DNA and mRNA in liver. Both LDLR protein and mRNA were detectable 1 day after dosing and kept increasing until d7–d14 (Figures 6C and 6D). Both IHC and ISH signals were significantly reduced on d120, consistent with the qPCR results (Figure 6B).

DISCUSSION

The successful clinical trial of liver-directed gene transfer for hemophilia B[34] paved the way for several clinical studies with AAV liver-directed gene transfer in recent years. It has been demonstrated that not only plasma protein deficiencies but also metabolic disorders can be treated with liver-directed gene therapy.[35] However, the potential clinical ramifications of AAV gene therapy should be approached very cautiously. Dose-correlated efficacy, host immune response, and toxicity have been reported in several clinical trials in which AAV vector was delivered systemically.[36,37] All three patients in cohort 2 [7.5×10^{12} GC/kg] of the first AAV8-mediated h*LDLR* gene transfer trial in HoFH patients (ClinicalTrials.gov: NCT02651675) experienced an asymptomatic elevation in transaminases 4–6 weeks post-dosing.[17] For genetic diseases such as HoFH, an ideal gene therapy vector should be able to achieve sustained therapeutic effects at a low or tolerable vector dose without eliciting harmful immune responses or toxicity. The combination of an AAV serotype with high transduction efficiency and an optimized vector construct could potentially achieve therapeutic effects at a safe vector dose. In this study, we aimed to develop a second-generation human LDLR transgene expression cassette with improved expression levels that could ultimately be used to treat HoFH patients.

Codon optimization has become a commonly used method to increase the expression of biotherapeutic recombinant proteins, such as antibodies. Increased expression of an antibody can be obtained by using synonymous codon mutations to fine-tune the expression of one of two light chain genes of a bispecific antibody.[38] Recently, it was shown that synonymous codon usage in open reading frame (ORF) sequences determines both gene expression levels and translation dynamics, suggesting codon usage influences gene expression.[39–41]

In 2011, Nathwani et al.[34] achieved sustained therapeutic expression of clotting factor IX in six human patients with severe hemophilia B by injecting a single dose of self-complementary AAV8 vector expressing a codon-optimized human factor IX transgene cDNA. Following this successful clinical trial, several pharmaceutical companies have used alternative AAV vector serotypes and different

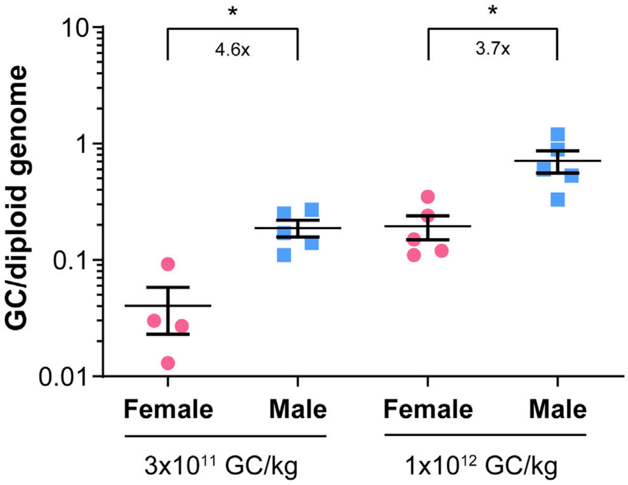

Figure 4. Gene transfer is more efficient in male DKO mice than in female DKO mice

Liver was harvested 28 days after AAV8.IVS2.hLDLR011-T vector administration at 3×10^{11} and 1×10^{12} GC/kg in female and male DKO mice for DNA isolation followed by qPCR. Individual mouse data and means ± SEM are shown. Statistical differences between female and male at each dose were analyzed by unpaired two-tailed Student's t test.

codon-optimized transgenes in many clinical trials. Similarly, we have developed variants of codon-optimized h*LDLR* cDNA sequences to achieve maximum expression at a low vector dosage. Our study highlighted that hLDLR011 is 3-fold more efficient than WT human *LDLR*. In a variety of organisms, transcriptional efficiency has been increased because of the occurrence of IVSs (or introns) at their 5′ UTR. One of the key early studies demonstrated that recombinant simian virus 40 carrying β-globin cDNA showed 400-fold increased RNA production in the presence of either β-globin IVS1 or IVS2 in their expression cassette compared with the intron-deficient transgene vector.[42] The inclusion of IVS2 or WPRE in the expression cassette increased expression of clotting factor IX, with this approach being employed effectively in AAV gene therapy vectors used to treat hemophilia B.[27,28] We have also demonstrated that the incorporation of IVS2 or WPRE increased the transgene expression several-fold compared with PI.hLDLR-WT or PI.hLDLR011.

In this study, we observed that at a low dose of 3×10^{11} GC/kg dose, male DKO mice treated with AAV8.IVS2.hLDLR011-T vector had about 0.2 vector genome copy per diploid genome but showed strong expression of hLDLR on the surface of transduced hepatocytes (Figure 3B), which resulted in 98% reduction of LDL-C 14 days after vector treatment (Figure 2C). This indicates that broad transduction and expression of LDLR in the liver may not be essential for achieving therapeutic effects in HoFH. Strong expression of LDLR in a few transduced hepatocytes could achieve dramatic therapeutic effects in a mouse model of HoFH.

We also observed the influence of sex on liver-directed AAV transduction efficiency in mice. In our study, female DKO mice had 4- to 5-fold lower vector genome copies in liver at the same doses. This impact of sex on liver transduction efficiency in mouse has been previously reported by other groups.[43–45] AAV transgene expression is reduced in male mice to levels seen in female mice following the castration of male mice, thereby suggesting that androgens play a vital role in transgene expression within hepatocytes.[43] However, the influence of sex on liver-directed gene transfer in nonhuman primates is not evident.[15]

LDLr$^{-/-}$ *Apobec*$^{-/-}$ DKO mice display a substantial increase of plasma LDL-C levels on standard chow and develop severe atherosclerosis, making this a more suited model to study the pathophysiology of human FH.[46] The DKO mice develop severe spontaneous atherosclerosis slowly and progressively on a standard chow diet as they age.[47] At the age of 12 weeks, a fatty streak develops in the proximal aortic region, and with age, plaque formation worsens. Severe plaque formation occurs by the age of 72 weeks, occupying over 60% of the arterial tree.[47] In this study, we observed very high serum LDL-C levels but did not observe any plaque formation as the study was executed in mice at the average age of 11 weeks. When DKO mice are fed the Western-type diet, they rapidly develop atherosclerosis at a very early age.[46] Our group has previously shown that the gene transfer of AAV8 vector expressing a murine form of LDLR reduced plasma cholesterol and showed complete regression of atherosclerosis (aortic plaque formation) despite the continuation of a Western-type diet throughout the study.[48] The current study focused on developing second-generation human LDLR vectors to achieve therapeutic effects at a low dose. Future studies could be conducted to examine whether AAV8-IVS2.hLDLR011-T and AAV8-IVS2.hLDLR011 effectively reduce atherosclerotic lesion progression in DKO mice fed a Western diet. The advantages of the LDLR triple variant in IVS2.hLDLR011-T can also be confirmed by performing studies in DKO mice overexpressing PCSK9 and/or IDOL.

To treat HoFH, long-term gene transfer is required in patients to effectively control hypercholesterolemia and atherosclerotic plaque formation. We have previously shown that the hepatocyte-targeted AAV8 vector with the liver-specific TBG promoter induces persistent and stable transgene expression for up to 180 days in DKO mice.[29] In the current study, serum LDL levels remained as low as 1% of pretreatment levels in DKO mice for 120 days following a single injection of 7.5×10^{12} GC/kg AAV8-IVS2.hLDLR011-T. Persistent transgene expression of AAV8-IVS2.hLDLR011-T was observed for 120 days in this study, although vector DNA and transgene expression levels reduced by ~4-fold compared with those in the peak time. Reduction of vector genome copies and/or transgene gene expression levels during the later phase compared with the early peak time has been observed in liver-directed gene therapy studies in animal models and in clinical trials,[10,13,26,49] which could be potentially attributed to the AAV vector biology, turnover of the transduced hepatocytes over time, or mechanisms related to host immune response. A long-term dose-response study in DKO mice can also be performed to investigate the role played by overexpression of LDLR. The findings

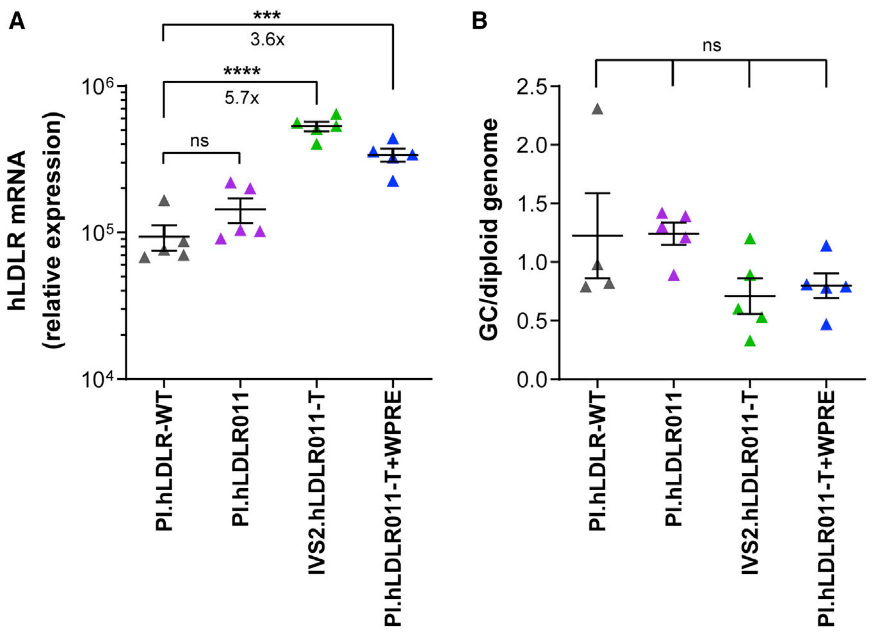

Figure 5. Vector optimization improves LDLR mRNA levels in male DKO mice

(A and B) Livers were harvested 28 days after AAV8.hLDLR vector administration at 1×10^{12} GC/kg in male DKO mice for RNA isolation followed by qRT-PCR (A) or DNA isolation for vector GC analysis by qPCR (B), respectively. Individual mouse data and means ± SEM are shown. ***p < 0.001, ****p < 0.0001, one-way ANOVA followed by Tukey's multiple comparison test.

presented here demonstrate that the combination of codon optimization and incorporation of IVS2 or WPRE enhanced transgene expression and reduced LDL-C levels more efficiently than the first generation AAV8-PI.hLDLR-WT vector. Based on our results, the second-generation clinical candidate vector we have developed, AAV8-IVS2.hLDLR011-T, is a promising vector to achieve therapeutic effects in HoFH patients.

MATERIALS AND METHODS

Codon optimization and vector construction and production

In order to improve the expression of h*LDLR*, we inserted a Kozak sequence (GCCACC) before the start codon of the original AAV construct containing the native h*LDLR* cDNA (referred to as hLDLR-WT).[50] Codon optimization was performed as described in the patent application WO2015012924. Specifically, codon bias tables B, D, and E from WO2015012924 were used to reverse-translate the amino acid sequence of LDLR. In addition, a separate codon bias table was created based on the native sequence of the macaque antibody IgG1-201[51] and applied to LDLR amino acid sequence to generate a new coding sequence. Four different codon-optimized cDNA sequences were synthesized and cloned into the AAV plasmids. GeneScript Rare Codon Analysis Tool (https://www.genscript.com/tools/rare-codon-analysis) was used to evaluate the properties of the hLDLR-WT and optimized h*LDLR* cDNA that include CAI, guanine-cytosine content (GC-content), and codon frequency distribution. Further vector optimization was performed on h*LDLR*011 by replacing the PI intron with IVS2 (Data S1) from the human beta-globin gene. h*LDLR*011-T was derived from hLDLR011 by site-directed mutagenesis using QuickChange Lightning kit (Agilent, Santa Clara, CA, USA) to incorporate the *LDLR* triple-mutant variant (hLDLR-L318D/K809R/C818A) to the hLDLR011 cDNA sequence (referred to as hLDLR011-T). WPRE (Data S1) was inserted at the 3′ end of the hLDLR-T cDNA. All AAV vectors were produced by triple transfection in 293 cells and the Penn Vector Core at the University of Pennsylvania as described previously.[52,53]

Animal studies

All animal studies were performed in accordance with protocols approved by Institutional Animal Care and Use Committee (IACUC) of the University of Pennsylvania. $Ldlr^{-/-}$, $Apobec1^{-/-}$ DKO mice were maintained on a chow diet at an animal facility in the University of Pennsylvania. DKO mice, aged 9–15 weeks (mean age, 11 weeks), were enrolled into the study. Vectors were injected intravenously via the tail vein at the specified dose. Blood was collected before vector dosing and at specified time points after dosing.

Serum non-HDL-C or LDL-C levels

Serum non-HDL-C levels were measured as previously described.[33] Serum LDL-C levels were measured by Antech GLP (Morrisville, NC, USA). Data after vector treatment are also presented as percentage of baseline levels.

Quantification of vector genomes and transgene mRNA in liver

Vector genomes in liver were quantified by qPCR (TaqMan Universal MasterMix; Applied Biosystems/Thermo Fisher Scientific, Waltham, MA, USA) as described previously.[54] RNA isolation and qRT-PCR were performed as described previously.[55]

IHC staining and ISH

IHC staining to detect hLDLR protein was performed as previously described.[48] ISH for hLDLR011 was performed as previously described using Z-shaped probe pairs binding to hLDLR011.[55]

Western blot analysis

Western analysis on proteins in liver lysate (20 μg/lane) was performed as previously described.[25] LDLR and beta-tubulin were detected with rabbit anti-LDLR antibody (ab52818; Abcam, Cambridge, MA, USA) and rabbit anti-beta-tubulin antibody (ab6046; Abcam), respectively. Bound primary antibody was detected with horseradish peroxidase-conjugated goat anti-rabbit IgG antibody (Thermo Fisher) and SuperSignal West Pico Chemiluminescence Substrate (Thermo Fisher).

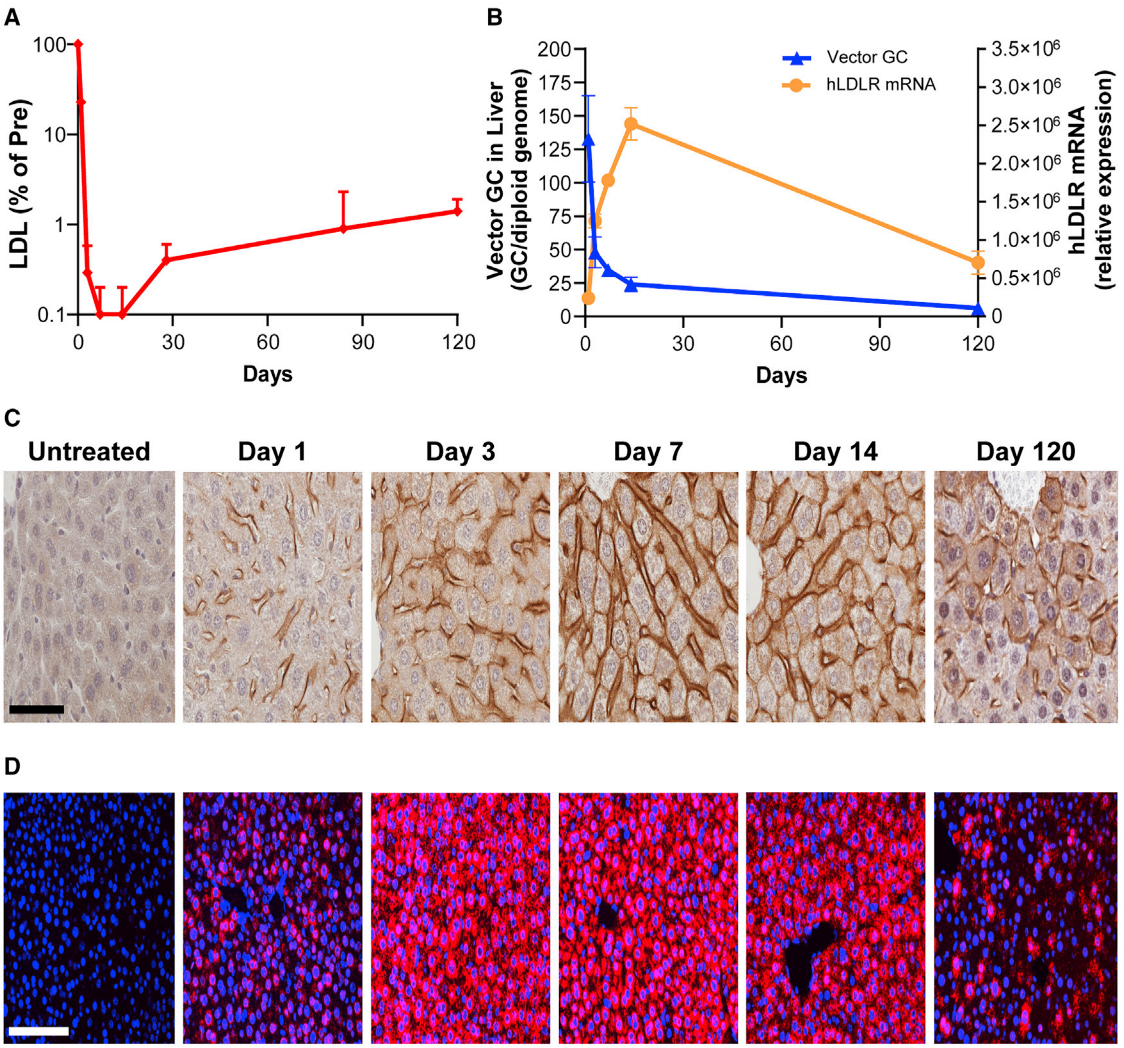

Figure 6. Kinetics of hLDLR expression in male DKO mice

Male DKO mice received 7.5 × 10^{12} GC/kg AAV8.IVS2.hLDLR-T via tail vein injection. Serum LDL-C levels were monitored over the course of the study. Livers were harvested from a cohort of five mice on d1, d3, d7, d14, and d120 for analysis. (A) Serum LDL-C levels. Means + SEM are shown (n = 5/time point). (B) Vector genome copies in liver measured by qPCR and hLDLR mRNA levels in liver measured by qRT-PCR. Means ± SEM are shown (n = 5/time point).(C) IHC to detect hLDLR protein. Scale bar, 50 μm. (D) ISH to detect vector DNA and hLDLR mRNA. Representative images are shown. Scale bar, 100 μm.

Statistical analyses

GraphPad Prism 9.0 was used for statistical analyses. An unpaired Student's t test was used for comparison of vector genome copies between females and males. Comparisons between multiple groups or multiple time points were performed using one-way analysis of variance (ANOVA) followed by Dunnett's multiple comparison test or Tukey's multiple comparison test. All values are expressed as mean ± SEM.

Data availability statement

All data and supporting materials are available within the article and Supplemental information.

SUPPLEMENTAL INFORMATION

Supplemental information can be found online at https://doi.org/10.1016/j.omtm.2021.04.017.

ACKNOWLEDGMENTS

We thank the Penn Vector Core for supplying AAV vectors, the Program in Comparative Medicine at the Gene Therapy Program (GTP) for animal care and procedures, and the Histology Core at GTP for histology analyses. We thank Nathan Denton for assistance with manuscript preparation and graphics. This work was funded by the National Institutes of Health, National Heart, Lung, and Blood Institute (P01 HL059407 to J.M.W.).

AUTHOR CONTRIBUTIONS

L.W., S.S., A.P.T., and J.M.W. conceived the project; L.W. and A.P.T. designed vector constructs; L.W., I.M., and S.S. designed the studies and analyzed the data; I.M., S.S., H.Z., P.B., Z.H., H.Y., and Y.Z. performed the experiments; L.W. and I.M. wrote the manuscript, which was revised and approved by all authors.

DECLARATION OF INTERESTS

J.M.W. is a paid advisor to and holds equity in Scout Bio and Passage Bio; he also has sponsored research agreements with Amicus Therapeutics, Biogen, Elaaj Bio, FA212, Janssen, Passage Bio, Regeneron, and Scout Bio, which are licensees of Penn technology. J.M.W., L.W., S.S., and A.P.T. are inventors on patents that have been licensed to various biopharmaceutical companies and for which they may receive payments.

REFERENCES

1. Cuchel, M., Bruckert, E., Ginsberg, H.N., Raal, F.J., Santos, R.D., Hegele, R.A., Kuivenhoven, J.A., Nordestgaard, B.G., Descamps, O.S., Steinhagen-Thiessen, E., et al.; European Atherosclerosis Society Consensus Panel on Familial Hypercholesterolaemia (2014). Homozygous familial hypercholesterolaemia: new insights and guidance for clinicians to improve detection and clinical management. A position paper from the Consensus Panel on Familial Hypercholesterolaemia of the European Atherosclerosis Society. Eur. Heart J. *35*, 2146–2157.
2. Nordestgaard, B.G., Chapman, M.J., Humphries, S.E., Ginsberg, H.N., Masana, L., Descamps, O.S., Wiklund, O., Hegele, R.A., Raal, F.J., Defesche, J.C., et al.; European Atherosclerosis Society Consensus Panel (2013). Familial hypercholesterolaemia is underdiagnosed and undertreated in the general population: guidance for clinicians to prevent coronary heart disease: consensus statement of the European Atherosclerosis Society. Eur. Heart J. *34*, 3478–3490a.
3. Goldstein, J.L., and Brown, M.S. (1995). Familial hypercholesterolemia. In The Metabolic Basis of Inherited Disease, Seventh Edition, C.R. Scriver, A.C. Beaudet, W.S. Sly, and D. Valle, eds. (McGraw Hill Book Co.), pp. 1981–2030.
4. Thompson, G.R. (2013). The evidence-base for the efficacy of lipoprotein apheresis in combating cardiovascular disease. Atheroscler. Suppl. *14*, 67–70.
5. Page, M.M., Ekinci, E.I., Jones, R.M., Angus, P.W., Gow, P.J., and O'Brien, R.C. (2014). Liver transplantation for the treatment of homozygous familial hypercholesterolaemia in an era of emerging lipid-lowering therapies. Intern. Med. J. *44*, 601–604.
6. Harada-Shiba, M., Ikewaki, K., Nohara, A., Otsubo, Y., Yanagi, K., Yoshida, M., Chang, Q., and Foulds, P. (2017). Efficacy and Safety of Lomitapide in Japanese Patients with Homozygous Familial Hypercholesterolemia. J. Atheroscler. Thromb. *24*, 402–411.
7. Robinson, J.G., Farnier, M., Krempf, M., Bergeron, J., Luc, G., Averna, M., Stroes, E.S., Langslet, G., Raal, F.J., El Shahawy, M., et al.; ODYSSEY LONG TERM Investigators (2015). Efficacy and safety of alirocumab in reducing lipids and cardiovascular events. N. Engl. J. Med. *372*, 1489–1499.
8. Blom, D.J., Harada-Shiba, M., Rubba, P., Gaudet, D., Kastelein, J.J.P., Charng, M.J., Pordy, R., Donahue, S., Ali, S., Dong, Y., et al. (2020). Efficacy and Safety of Alirocumab in Adults With Homozygous Familial Hypercholesterolemia: The ODYSSEY HoFH Trial. J. Am. Coll. Cardiol. *76*, 131–142.
9. Van Craeyveld, E., Jacobs, F., Gordts, S.C., and De Geest, B. (2011). Gene therapy for familial hypercholesterolemia. Curr. Pharm. Des. *17*, 2575–2591.
10. Nathwani, A.C., Reiss, U.M., Tuddenham, E.G., Rosales, C., Chowdary, P., McIntosh, J., Della Peruta, M., Lheriteau, E., Patel, N., Raj, D., et al. (2014). Long-term safety and efficacy of factor IX gene therapy in hemophilia B. N. Engl. J. Med. *371*, 1994–2004.
11. George, L.A., Sullivan, S.K., Giermasz, A., Rasko, J.E.J., Samelson-Jones, B.J., Ducore, J., Cuker, A., Sullivan, L.M., Majumdar, S., Teitel, J., et al. (2017). Hemophilia B Gene Therapy with a High-Specific-Activity Factor IX Variant. N. Engl. J. Med. *377*, 2215–2227.
12. Miesbach, W., Meijer, K., Coppens, M., Kampmann, P., Klamroth, R., Schutgens, R., Tangelder, M., Castaman, G., Schwäble, J., Bonig, H., et al. (2018). Gene therapy with adeno-associated virus vector 5-human factor IX in adults with hemophilia B. Blood *131*, 1022–1031.
13. Pasi, K.J., Rangarajan, S., Mitchell, N., Lester, W., Symington, E., Madan, B., Laffan, M., Russell, C.B., Li, M., Pierce, G.F., and Wong, W.Y. (2020). Multiyear Follow-up of AAV5-hFVIII-SQ Gene Therapy for Hemophilia A. N. Engl. J. Med. *382*, 29–40.
14. Greig, J.A., Limberis, M.P., Bell, P., Chen, S.J., Calcedo, R., Rader, D.J., and Wilson, J.M. (2017). Nonclinical Pharmacology/Toxicology Study of AAV8.TBG.mLDLR and AAV8.TBG.hLDLR in a Mouse Model of Homozygous Familial Hypercholesterolemia. Hum. Gene Ther. Clin. Dev. *28*, 28–38.
15. Greig, J.A., Limberis, M.P., Bell, P., Chen, S.J., Calcedo, R., Rader, D.J., and Wilson, J.M. (2017). Non-Clinical Study Examining AAV8.TBG.hLDLR Vector-Associated Toxicity in Chow-Fed Wild-Type and $LDLR^{+/-}$ Rhesus Macaques. Hum. Gene Ther. Clin. Dev. *28*, 39–50.
16. Regenxbio Inc.. A Gene Therapy Study for Homozygous Familial Hypercholesterolemia (HoFH). https://clinicaltrials.gov/ct2/show/NCT02651675.
17. Cuchel, M., Bajaj, A., Carr, R., Sikora, T., Duell, P.B., Tardif, J.-C., Roeters van Lennep, J.E., Linton, M.F., Averna, M., Cho, Y., et al. (2020). Use of Prophylactic Steroids to Mitigate Potential T-Cell Response in AAV8-Mediated hLDLRGene Transfer in Subjects with Homozygous Familial Hypercholesterolemia. Mol. Ther. *28*, 271.
18. Goldstein, J.L., DeBose-Boyd, R.A., and Brown, M.S. (2006). Protein sensors for membrane sterols. Cell *124*, 35–46.
19. Zelcer, N., Hong, C., Boyadjian, R., and Tontonoz, P. (2009). LXR regulates cholesterol uptake through Idol-dependent ubiquitination of the LDL receptor. Science *325*, 100–104.
20. Dubuc, G., Chamberland, A., Wassef, H., Davignon, J., Seidah, N.G., Bernier, L., and Prat, A. (2004). Statins upregulate PCSK9, the gene encoding the proprotein convertase neural apoptosis-regulated convertase-1 implicated in familial hypercholesterolemia. Arterioscler. Thromb. Vasc. Biol. *24*, 1454–1459.
21. Costet, P., Hoffmann, M.M., Cariou, B., Guyomarc'h Delasalle, B., Konrad, T., and Winkler, K. (2010). Plasma PCSK9 is increased by fenofibrate and atorvastatin in a non-additive fashion in diabetic patients. Atherosclerosis *212*, 246–251.
22. Blom, D.J., Hala, T., Bolognese, M., Lillestol, M.J., Toth, P.D., Burgess, L., Ceska, R., Roth, E., Koren, M.J., Ballantyne, C.M., et al.; DESCARTES Investigators (2014). A 52-week placebo-controlled trial of evolocumab in hyperlipidemia. N. Engl. J. Med. *370*, 1809–1819.
23. Koren, M.J., Lundqvist, P., Bolognese, M., Neutel, J.M., Monsalvo, M.L., Yang, J., Kim, J.B., Scott, R., Wasserman, S.M., and Bays, H.; MENDEL-2 Investigators (2014). Anti-PCSK9 monotherapy for hypercholesterolemia: the MENDEL-2 randomized, controlled phase III clinical trial of evolocumab. J. Am. Coll. Cardiol. *63*, 2531–2540.
24. Somanathan, S., Jacobs, F., Wang, Q., Hanlon, A.L., Wilson, J.M., and Rader, D.J. (2014). AAV vectors expressing LDLR gain-of-function variants demonstrate increased efficacy in mouse models of familial hypercholesterolemia. Circ. Res. *115*, 591–599.
25. Wang, L., Wang, H., Morizono, H., Bell, P., Jones, D., Lin, J., McMenamin, D., Yu, H., Batshaw, M.L., and Wilson, J.M. (2012). Sustained correction of OTC deficiency in spf(ash) mice using optimized self-complementary AAV2/8 vectors. Gene Ther. *19*, 404–410.
26. Nathwani, A.C., Gray, J.T., Ng, C.Y., Zhou, J., Spence, Y., Waddington, S.N., Tuddenham, E.G., Kemball-Cook, G., McIntosh, J., Boon-Spijker, M., et al. (2006). Self-complementary adeno-associated virus vectors containing a novel liver-specific human factor IX expression cassette enable highly efficient transduction of murine and nonhuman primate liver. Blood *107*, 2653–2661.
27. Wang, L., Takabe, K., Bidlingmaier, S.M., Ill, C.R., and Verma, I.M. (1999). Sustained correction of bleeding disorder in hemophilia B mice by gene therapy. Proc. Natl. Acad. Sci. USA *96*, 3906–3910.
28. Wang, L., Nichols, T.C., Read, M.S., Bellinger, D.A., and Verma, I.M. (2000). Sustained expression of therapeutic level of factor IX in hemophilia B dogs by AAV-mediated gene therapy in liver. Mol. Ther. *1*, 154–158.

29. Chen, S.J., Sanmiguel, J., Lock, M., McMenamin, D., Draper, C., Limberis, M.P., Kassim, S.H., Somanathan, S., Bell, P., Johnston, J.C., et al. (2013). Biodistribution of AAV8 vectors expressing human low-density lipoprotein receptor in a mouse model of homozygous familial hypercholesterolemia. Hum. Gene Ther. Clin. Dev. *24*, 154–160.

30. Powell, S.K., Rivera-Soto, R., and Gray, S.J. (2015). Viral expression cassette elements to enhance transgene target specificity and expression in gene therapy. Discov. Med. *19*, 49–57.

31. Loeb, J.E., Cordier, W.S., Harris, M.E., Weitzman, M.D., and Hope, T.J. (1999). Enhanced expression of transgenes from adeno-associated virus vectors with the woodchuck hepatitis virus posttranscriptional regulatory element: implications for gene therapy. Hum. Gene Ther. *10*, 2295–2305.

32. Lipshutz, G.S., Titre, D., Brindle, M., Bisconte, A.R., Contag, C.H., and Gaensler, K.M. (2003). Comparison of gene expression after intraperitoneal delivery of AAV2 or AAV5 in utero. Mol. Ther. *8*, 90–98.

33. Kassim, S.H., Li, H., Bell, P., Somanathan, S., Lagor, W., Jacobs, F., Billheimer, J., Wilson, J.M., and Rader, D.J. (2013). Adeno-associated virus serotype 8 gene therapy leads to significant lowering of plasma cholesterol levels in humanized mouse models of homozygous and heterozygous familial hypercholesterolemia. Hum. Gene Ther. *24*, 19–26.

34. Nathwani, A.C., Tuddenham, E.G., Rangarajan, S., Rosales, C., McIntosh, J., Linch, D.C., Chowdary, P., Riddell, A., Pie, A.J., Harrington, C., et al. (2011). Adenovirus-associated virus vector-mediated gene transfer in hemophilia B. N. Engl. J. Med. *365*, 2357–2365.

35. Ginocchio, V.M., Ferla, R., Auricchio, A., and Brunetti-Pierri, N. (2019). Current Status on Clinical Development of Adeno-Associated Virus-Mediated Liver-Directed Gene Therapy for Inborn Errors of Metabolism. Hum. Gene Ther. *30*, 1204–1210.

36. Manno, C.S., Pierce, G.F., Arruda, V.R., Glader, B., Ragni, M., Rasko, J.J., Ozelo, M.C., Hoots, K., Blatt, P., Konkle, B., et al. (2006). Successful transduction of liver in hemophilia by AAV-Factor IX and limitations imposed by the host immune response. Nat. Med. *12*, 342–347.

37. Chand, D., Mohr, F., McMillan, H., Tukov, F.F., Montgomery, K., Kleyn, A., Sun, R., Tauscher-Wisniewski, S., Kaufmann, P., and Kullak-Ublick, G. (2021). Hepatotoxicity following administration of onasemnogene abeparvovec (AVXS-101) for the treatment of spinal muscular atrophy. J Hepatol. *74*, 560–566.

38. Magistrelli, G., Poitevin, Y., Schlosser, F., Pontini, G., Malinge, P., Josserand, S., Corbier, M., and Fischer, N. (2017). Optimizing assembly and production of native bispecific antibodies by codon de-optimization. MAbs *9*, 231–239.

39. Zhou, Z., Dang, Y., Zhou, M., Li, L., Yu, C.H., Fu, J., Chen, S., and Liu, Y. (2016). Codon usage is an important determinant of gene expression levels largely through its effects on transcription. Proc. Natl. Acad. Sci. USA *113*, E6117–E6125.

40. Newman, Z.R., Young, J.M., Ingolia, N.T., and Barton, G.M. (2016). Differences in codon bias and GC content contribute to the balanced expression of TLR7 and TLR9. Proc. Natl. Acad. Sci. USA *113*, E1362–E1371.

41. Presnyak, V., Alhusaini, N., Chen, Y.H., Martin, S., Morris, N., Kline, N., Olson, S., Weinberg, D., Baker, K.E., Graveley, B.R., and Coller, J. (2015). Codon optimality is a major determinant of mRNA stability. Cell *160*, 1111–1124.

42. Buchman, A.R., and Berg, P. (1988). Comparison of intron-dependent and intron-independent gene expression. Mol. Cell. Biol. *8*, 4395–4405.

43. Davidoff, A.M., Ng, C.Y., Zhou, J., Spence, Y., and Nathwani, A.C. (2003). Sex significantly influences transduction of murine liver by recombinant adeno-associated viral vectors through an androgen-dependent pathway. Blood *102*, 480–488.

44. Dane, A.P., Cunningham, S.C., Graf, N.S., and Alexander, I.E. (2009). Sexually dimorphic patterns of episomal rAAV genome persistence in the adult mouse liver and correlation with hepatocellular proliferation. Mol. Ther. *17*, 1548–1554.

45. Guenzel, A.J., Collard, R., Kraus, J.P., Matern, D., and Barry, M.A. (2015). Long-term sex-biased correction of circulating propionic acidemia disease markers by adeno-associated virus vectors. Hum. Gene Ther. *26*, 153–160.

46. Powell-Braxton, L., Véniant, M., Latvala, R.D., Hirano, K.I., Won, W.B., Ross, J., Dybdal, N., Zlot, C.H., Young, S.G., and Davidson, N.O. (1998). A mouse model of human familial hypercholesterolemia: markedly elevated low density lipoprotein cholesterol levels and severe atherosclerosis on a low-fat chow diet. Nat. Med. *4*, 934–938.

47. Miyajima, C., Iwaki, T., Umemura, K., Ploplis, V.A., and Castellino, F.J. (2018). Characterization of Atherosclerosis Formation in a Murine Model of Type IIa Human Familial Hypercholesterolemia. BioMed Res. Int. *2018*, 1878964.

48. Kassim, S.H., Li, H., Vandenberghe, L.H., Hinderer, C., Bell, P., Marchadier, D., Wilson, A., Cromley, D., Redon, V., Yu, H., et al. (2010). Gene therapy in a humanized mouse model of familial hypercholesterolemia leads to marked regression of atherosclerosis. PLoS ONE *5*, e13424.

49. Nakai, H., Fuess, S., Storm, T.A., Muramatsu, S., Nara, Y., and Kay, M.A. (2005). Unrestricted hepatocyte transduction with adeno-associated virus serotype 8 vectors in mice. J. Virol. *79*, 214–224.

50. Lebherz, C., Gao, G., Louboutin, J.P., Millar, J., Rader, D., and Wilson, J.M. (2004). Gene therapy with novel adeno-associated virus vectors substantially diminishes atherosclerosis in a murine model of familial hypercholesterolemia. J. Gene Med. *6*, 663–672.

51. Glamann, J., and Hirsch, V.M. (2000). Characterization of a macaque recombinant monoclonal antibody that binds to a CD4-induced epitope and neutralizes simian immunodeficiency virus. J. Virol. *74*, 7158–7163.

52. Lock, M., Alvira, M., Vandenberghe, L.H., Samanta, A., Toelen, J., Debyser, Z., and Wilson, J.M. (2010). Rapid, simple, and versatile manufacturing of recombinant adeno-associated viral vectors at scale. Hum. Gene Ther. *21*, 1259–1271.

53. Lock, M., Alvira, M.R., Chen, S.J., and Wilson, J.M. (2014). Absolute determination of single-stranded and self-complementary adeno-associated viral vector genome titers by droplet digital PCR. Hum. Gene Ther. Methods *25*, 115–125.

54. Bell, P., Moscioni, A.D., McCarter, R.J., Wu, D., Gao, G., Hoang, A., Sanmiguel, J.C., Sun, X., Wivel, N.A., Raper, S.E., et al. (2006). Analysis of tumors arising in male B6C3F1 mice with and without AAV vector delivery to liver. Mol. Ther. *14*, 34–44.

55. Wang, L., Smith, J., Breton, C., Clark, P., Zhang, J., Ying, L., Che, Y., Lape, J., Bell, P., Calcedo, R., et al. (2018). Meganuclease targeting of PCSK9 in macaque liver leads to stable reduction in serum cholesterol. Nat. Biotechnol. *36*, 717–725.

OMTM, Volume *22*

Supplemental information

Developing a second-generation clinical candidate AAV vector for gene therapy of familial hypercholesterolemia

Lili Wang, Ilayaraja Muthuramu, Suryanarayan Somanathan, Hong Zhang, Peter Bell, Zhenning He, Hongwei Yu, Yanqing Zhu, Anna P. Tretiakova, and James M. Wilson

Table S1. Significance of reduction of serum LDL-C levels in female and male DKO mice on days 14 and 28 after administration of AAV8.hLDLR vectors at different doses.

Vector	F, 3x10^{11} GC/kg		F, 1x10^{12} GC/kg		M, 1x10^{11} GC/kg		M, 3x10^{11} GC/kg		M, 1x10^{12} GC/kg	
(n=5)	d14	d28	d14	d28	d14	d28	d14	d28	d14	d28
PI.hLDLR-WT	ns	(**)	**	*	****	***	*	**	****	****
PI.hLDLR011	ns	ns	**	**	**	***	****	****	****	****
IVS2.hLDLR011	ns	ns	***	***	****	****	***	***	***	***
IVS2.hLDLR011-T	ns	ns	***	***	****	***	****	****	****	****
PI.hLDLR011-T +WPRE	ns	ns	***	**	****	****	****	****	****	****

Serum LDL-C levels on days -1 (d-1), 14 (d14), and 28 (d28) are shown in Figure 2C. GraphPad Prism 9.0 was used for statistical analyses. For comparisons of LDL-C before (d-1) and after vector treatment (d14 and d28), RM one-way ANOVA with Geisser-Greenhouse correction was applied, with Dunnett's multiple comparison test to test for significant differences on d14 and d28 after vector treatment. * $p<0.05$, ** $p<0.01$, *** $p<0.001$, ****$p<0.0001$. (**) indicates d28 levels were significantly higher than d-1 levels.

Table S2. Comparison of the level of serum LDL-C reduction in female and male DKO mice treated with different AAV8.hLDLR vectors.

Day 14	Vector	PBS	PI.hLDLR-WT	PI.hLDLR011	IVS2.hLDLR011	IVS2.hLDLR011-T
Female DKO	PBS					
	PI.hLDLR-WT	ns				
	PI.hLDLR011	ns	ns			
$3x10^{11}$ GC/kg	IVS2.hLDLR011	*	*	ns		
	IVS2.hLDLR011-T	ns	ns	ns	ns	
	PI.hLDLR011-T+WPRE	ns	ns	ns	ns	ns

Day 28	Vector	PBS	PI.hLDLR-WT	PI.hLDLR011	IVS2.hLDLR011	IVS2.hLDLR011-T
Female DKO	PBS					
	PI.hLDLR-WT	ns				
	PI.hLDLR011	ns	ns			
$3x10^{11}$ GC/kg	IVS2.hLDLR011	***	****	*		
	IVS2.hLDLR011-T	*	**	ns	ns	
	PI.hLDLR011-T+WPRE	ns	ns	ns	**	ns

Day 14	Vector	PBS	PI.hLDLR-WT	PI.hLDLR011	IVS2.hLDLR011	IVS2.hLDLR011-T
Female DKO	PBS					
	PI.hLDLR-WT	****				
	PI.hLDLR011	****	ns			
$1x10^{12}$ GC/kg	IVS2.hLDLR011	****	***	ns		
	IVS2.hLDLR011-T	****	**	ns	ns	
	PI.hLDLR011-T+WPRE	****	**	ns	ns	ns

Day 28	Vector	PBS	PI.hLDLR-WT	PI.hLDLR011	IVS2.hLDLR011	IVS2.hLDLR011-T
Female DKO	PBS					
	PI.hLDLR-WT	***				
	PI.hLDLR011	****	ns			
$1x10^{12}$ GC/kg	IVS2.hLDLR011	****	***	*		
	IVS2.hLDLR011-T	****	***	ns	ns	
	PI.hLDLR011-T+WPRE	****	*	ns	ns	ns

Day 14	Vector	PBS	PI.hLDLR-WT	PI.hLDLR011	IVS2.hLDLR011	IVS2.hLDLR011-T
Male DKO	PBS					
	PI.hLDLR-WT	***				
	PI.hLDLR011	****	****			
$1x10^{11}$ GC/kg	IVS2.hLDLR011	****	****	****		
	IVS2.hLDLR011-T	****	****	****	ns	
	PI.hLDLR011-T+WPRE	****	****	****	ns	ns

Day 28	Vector	PBS	PI.hLDLR-WT	PI.hLDLR011	IVS2.hLDLR011	IVS2.hLDLR011-T
Male DKO	PBS					
	PI.hLDLR-WT	****				
	PI.hLDLR011	****	**			
$1x10^{11}$ GC/kg	IVS2.hLDLR011	****	****	****		
	IVS2.hLDLR011-T	****	****	**	ns	
	PI.hLDLR011-T+WPRE	****	****	****	ns	ns

Day 14	Vector	PBS	PI.hLDLR-WT	PI.hLDLR011	IVS2.hLDLR011	IVS2.hLDLR011-T

Male DKO	PBS					
	PI.hLDLR-WT	****				
$3x10^{11}$ GC/kg or $1x10^{12}$ GC/kg	PI.hLDLR011	****	****			
	IVS2.hLDLR011	****	****	ns		
	IVS2.hLDLR011-T	****	****	ns	ns	
	PI.hLDLR011-T+WPRE	****	****	ns	ns	ns

Day 28	Vector					
Male DKO	PBS					
	PI.hLDLR-WT	****				
$3x10^{11}$ GC/kg or $1x10^{12}$ GC/kg	PI.hLDLR011	****	****			
	IVS2.hLDLR011	****	****	ns		
	IVS2.hLDLR011-T	****	****	ns	ns	
	PI.hLDLR011-T+WPRE	****	****	ns	ns	ns

Serum LDL-C levels on days 14 and 28 were normalized by baseline levels on day -1 and are shown in Figure 2D. GraphPad Prism 9.0 was used for statistical analyses. For comparisons of efficiency of different vectors at the specified dose, time point and sex of mice, one-way ANOVA was applied followed by Tukey's multiple comparison test. * $p<0.05$, ** $p<0.01$, *** $p<0.001$, ****$p<0.0001$. ns, not significant.

A

	GC content (%)	CAI	Rare codon percentage
hLDLR-WT	58.38	0.83	1
hLDLR-kozak	58.41	0.83	1
hLDLR001	67.02	0.97	0
hLDLR011	60.70	0.95	0
hLDLR201	62.66	0.96	0
hLDLR026	62.16	0.91	0

B

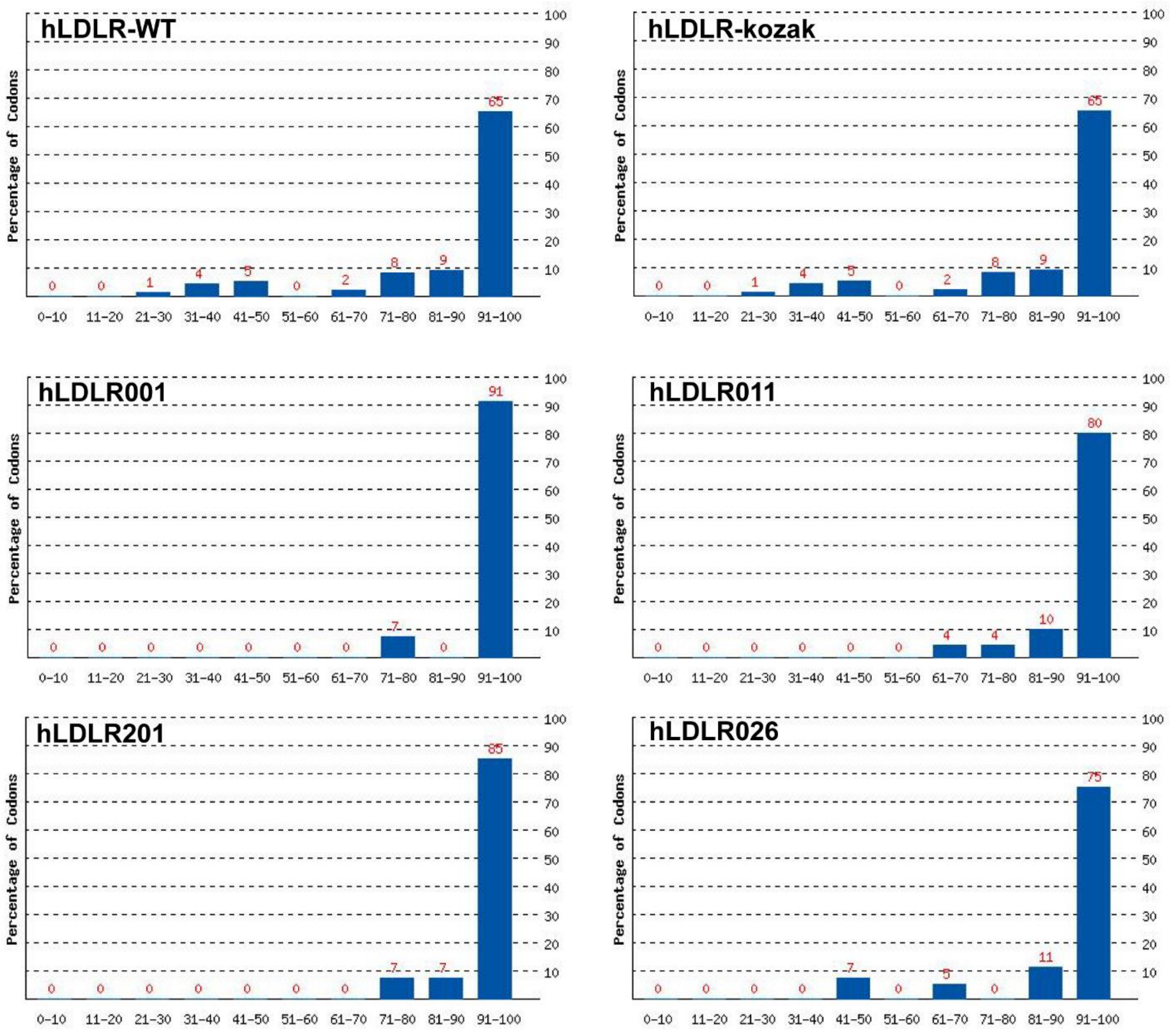

Figure S1. Rare codon analysis on the hLDLR cDNA sequences by GeneScript Rare Codon Analysis Tool. (A) GC content, CAI score, and rare codon percentage in the hLDLR cDNA sequences. (B) Analysis of codon frequency distribution. The value of 100 is set for the codon with highest usage frequency for a given amino acid in the host expression organism (human). Codons with values lower than 30 are considered to hinder protein expression.

Data file S1: Sequences for IVS2 and WPRE.

FASTA sequences:

>IVS2 572 bp

AGCTTACTTGTGGTACCGAGCTCGGATCCTGAGAACTTCAGGGTGAGTCTATGGGACCCTTG
ATGTTTTCTTTCCCCTTCTTTTCTATGGTTAAGTTCATGTCATAGGAAGGGGAGAAGTAACAG
GGTACACATATTGACCAAATCAGGGTAATTTTGCATTTGTAATTTTAAAAAATGCTTTCTTCT
TTTAATATACTTTTTTGTTTATCTTATTTCTAATACTTTCCCTAATCTCTTTCTTTCAGGGCAAT
AATGATACAATGTATCATGCCTCTTTGCACCATTCTAAAGAATAACAGTGATAATTTCTGGG
TTAAGGCAATAGCAATATTTCTGCATATAAATATTTCTGCATATAAATTGTAACTGATGTAA
GAGGTTTCATATTGCTAATAGCAGCTACAATCCAGCTACCATTCTGCTTTTATTTTATGGTTG
GGATAAGGCTGGATTATTCTGAGTCCAAGCTAGGCCCTTTTGCTAATCATGTTCATACCTCTT
ATCTTCCTCCCACAGCTCCTGGGCAACGTGCTGGTCTGTGTGCTGGCCCATCACTTTGGCAAA
GAATTG

>WPRE 542 bp

AATCAACCTCTGGATTACAAAATTTGTGAAAGATTGACTGGTATTCTTAACTATGTTGCTCCT
TTTACGCTATGTGGATACGCTGCTTTAATGCCTTTGTATCATGCTATTGCTTCCCGTATGGCTT
TCATTTTCTCCTCCTTGTATAAATCCTGGTTGCTGTCTCTTTATGAGGAGTTGTGGCCCGTTGT
CAGGCAACGTGGCGTGGTGTGCACTGTGTTTGCTGACGCAACCCCCACTGGTTGGGGCATTG
CCACCACCTGTCAGCTCCTTTCCGGGACTTTCGCTTTCCCCCTCCCTATTGCCACGGCGGAAC
TCATCGCCGCCTGCCTTGCCCGCTGCTGGACAGGGGCTCGGCTGTTGGGCACTGACAATTCC
GTGGTGTTGTCGGGGAAATCATCGTCCTTTCCTTGGCTGCTCGCCTGTGTTGCCACCTGGATT
CTGCGCGGGACGTCCTTCTGCTACGTCCCTTCGGCCCTCAATCCAGCGGACCTTCCTTCCCGC
GGCCTGCTGCCGGCTCTGCGGCCTCTTCCGCGTCTTCG
